# Breeding for higher yield, early maturity, wider adaptability and waterlogging tolerance in soybean (*Glycine max* L.): A case study

**DOI:** 10.1038/s41598-021-02064-x

**Published:** 2021-11-24

**Authors:** Shivakumar Maranna, Vennampally Nataraj, Giriraj Kumawat, Subhash Chandra, Vangala Rajesh, Rajkumar Ramteke, Ram Manohar Patel, Milind B. Ratnaparkhe, S. M. Husain, Sanjay Gupta, Nita Khandekar

**Affiliations:** ICAR-Indian Institute of Soybean Research, Indore, 452001 India

**Keywords:** Biological techniques, Genetics, Plant sciences

## Abstract

Breeding for higher yield and wider adaptability are major objectives of soybean crop improvement. In the present study, 68 advanced breeding lines along with seven best checks were evaluated for yield and attributing traits by following group balanced block design. Three blocks were constituted based on the maturity duration of the breeding lines. High genetic variability for the twelve quantitative traits was found within and across the three blocks. Several genotypes were found to outperform check varieties for yield and attributing traits. During the same crop season, one of the promising entries, NRC 128,was evaluated across seven locations for its wider adaptability and it has shown stable performance in Northern plain Zone with > 20% higher yield superiority over best check PS 1347. However, it produced 9.8% yield superiority over best check in Eastern Zone. Screening for waterlogging tolerance under artificial conditions revealed that NRC 128 was on par with the tolerant variety JS 97–52. Based on the yield superiority, wider adaptability and waterlogging tolerance, NRC 128 was released and notified by Central Varietal Release Committee (CVRC) of India, for its cultivation across Eastern and Northern Plain Zones of India.

## Introduction

Soybean (*Glycine max* (L.) Merril) being the world’s most important seed legume, has a prominent place among modern agricultural commodities. It contributes about 25% to the global edible oil production, about two thirds of the world’s protein concentrate for livestock feeding and is a valuable ingredient in formulated feeds for poultry and fish. It is also an important raw material for food, pharma and other industries. As per AMIS, FAO estimates, among the major soybean growing countries, India ranks fourth in terms of area and fifth in terms of production. During 2020–21, soybean was grown in an area of 12.06 million hectare with a production of 13.58 million tons and productivity of 1126 kg/ha. Indian soybean productivity is stagnated around 1200 kg/ha while world soybean productivity stands at 2900 kg/ha and the USA and Brazil are the countries with highest productivity (> 3000 kg /ha) (AMIS, FAO website). Lower soybean productivity in India is attributed to (1) rainfed nature and short duration (90–100 days) of the crop as compared to USA (160–170 days), (2) Emergence of biotic stresses particularly anthracnose, charcoal rot, and Rhizactonia aerial blight, and abiotic stresses like drought and waterlogging from past few years, and (3) narrow genetic base of the released cultivars and smaller F_2_ population size being used to identify the desirable segregants^1^. In any crop improvement program, breeders often keep in view a set of traits which, when brought together into a genotype would lead to high performance and such genotype is termed as ideotype^2^. The idea behind ideotype-design is to increase crop performance through the selection of genotypes based on multiple traits simultaneously^3^. Smith-Hazel (SH) index is a linear selection index that has been widely employed by breeders for multi-trait selection^4,5^. However, presence of multicollinearity and difficulty in assigning economic weightage to the traits under consideration in case of SH index can affect the genetic gain^3^. Therefore, to overcome these weaknesses, a multivariate selection index genotype–ideotype distance index (MGIDI) has been developed^3^ that accounts for the multicollinearity issue and selects all the traits under consideration favorably, thus resulting in significant genetic gain.

While dealing with quantitative and complex traits such as grain yield, effects of G × E interactions needs to be considered for genotypic evaluation and varietal selection^6,7^. Various stability models have been developed to understand the G × E interaction patterns^8,9^. Interactions become complex as the number of environments and genotypes increase, and detail analysis and understanding cannot be possible without a graphic approach. GGE (Genotype main effect (G) plus genotype by environment interaction (GE)) is a multivariate, graphic based stability model that has been extensively employed in stability analysis and in understanding Genotype × Environment Interactions, more commonly for grain yield^10^.

Waterlogging is a major abiotic stress significantly affecting world soybean production, causing 16% yield loss globally^11,12^and 18% yield loss in India^13^. Further, global climate change-based weather simulation models showed an expected increase in loss of crop production due to flooding in near future^14^. During last five cropping seasons, major soybean growing regions of central India received more than 70% rainfall during August–September months, when the crop is at late vegetative stage or early reproductive stage in farmer fields^15^, indicating the potential threat of waterlogging stress to the soybean production. Importance of breeding for waterlogging tolerance has been reported in India^15^. JS 20–38 an advanced breeding line has been identified as potential donor for the waterlogging tolerance^16^.Genome-wide association mapping of waterlogging tolerance has identified large number of favorable flood-tolerant alleles and new genetic sources for use in soybean breeding for waterlogging tolerance^17^.Till date only one waterlogging tolerant variety JS 97–52 is notified for cultivation in central zone and north-eastern zone of India. Utilizing this variety in the breeding program, some varieties JS 20–29, JS 20–69 and JS 20–98 were developed with objective of yield traits which were released for cultivation in Central Zone of India. So, there is a need to develop the variety which is having wider adaptability with waterlogging tolerance for other zones. In India, soybean crop especially in Eastern Zone comprising of Bihar, Chhattisgarh, Ranchi states affected by waterlogging conditions due to prolonged monsoon rains. In our earlier studies conducted at ICAR-IISR, cultivar JS 97–52 has been reported as waterlogging tolerant genotype and it is being used as tolerant check in evaluation studies conducted in India^18,19^. A very few researchers evaluated Indian soybean genotypes for waterlogging tolerance either at vegetative or reproductive stage^20^ but not at both stages. Keeping in view of soybean improvement under changing climate, the present study was undertaken to develop and evaluate several diverse breeding materials for identifying near-ideotype having higher yield potential, wider adaptability and waterlogging tolerance (Table 1).

**Table 1:** List of genotypes along with pedigree used in the evaluation for yield and attributing traits.

Sl. no	Genotypes	Pedigree
G1	3A-44–1-3	Type 49 × EC 538,828
G2	6A-47–1	JS 335 × EC 538,828
G3	3A-60–6	Type 49 × EC 538,828
G4	3A-17–1-8	Type 49 × EC 538,828
G5	3A-60–2	Type 49 × EC 538,828
G6	BC_3_F_4_(JS 95–60)-2	JS 95–60 × *G. soja*
G7	6A-34–6	JS 335 × EC 538,828
C1	JS 20–34 (C)	JS 98–63 × PK 768
C2	JS 95–60 (C)	Selection from PS 73–22
G8	3A-17–1-2	Type 49 × EC 538,828
G9	EC 572,109	Germplasm collection
G10	12–96	JS 20–38 × JS 335
G11	12–22	JS 335 × AGS 191
G12	3A-93–1-2	Type 49 × EC 538,828
G13	7A-68–1	JS335 x EC 538,828
G14	12–16	JS 335 × AGS 191
G15	8–24-2	JS 97–52 × EC 538,828
G16	3A-44–1-1	Type 49 × EC 538,828
G17	13–2	NRC 86 × MACS 330
G18	IC 15,089	Indigenous germplasm collection
G19	8–98-1	JS 97–52 × EC 538,828
G20	6A-34–11 (NRC 146)	JS 335 × EC 538,828
G21	8–101-3	JS 97–52 × EC 538,828
G22	6A-47–4	JS 335 × EC 538,828
G23	8–24-3	JS 97–52 × EC 538,828
C3	JS 93–05	Secondary selectionfrom PS 73–22
G24	13–71	JS 97–52 × JS 335
G25	14–52	Bragg x JS 335
C4	JS 20–29 (C)	JS 97–52 × JS 95–56
G26	13–100	JS 97–52 × JS 335
G27	6A-34–12	JS 335 × EC 538,828
G28	15–64	Hardee x JS 335
G29	11–92	Doko x JS 335
G30	11–147	JS 335 × AGS 191
G31	6A-34–25	JS 335 × EC 538,828
G32	8–94-3	JS 97–52 × EC 538,828
G33	12–108	JS 20–38 × JS 335
G34	15–77	G11 x JS 335
G35	15–72	Hardee x JS 335
G36	13–150	JS 335 × JS 97–52
G37	6A-18–3-5	JS 335 × EC 538,828
G38	6A-33–1-2	JS 335 × EC 538,828
G39	232 D	JS 335 × *G. soja*
G40	15–137	JS 335 × AGS 191
G41	218 D	JS 335 × *G. soja*
G42	6A-18–3-1	JS 335 × EC 538,828
G43	15–46	Kalitur x JS 335
G44	15–1	JS 335 × MLT 1
G45	518 L	JS 335 × G. soja
C5	NRC 86 (C)	RKS 15 × EC 481,309
G46	8–66-2	JS 97–52 × EC 538,828
C6	JS 97–52	PK 327 × L129
G47	10–23-2–1 SP	Type 49 × JS 335
G48	14–74	Bragg x JS 335
G49	14–77	Bragg x JS 335
G50	NRC86 BC34	NRC 86 × *G. soja*
G51	14–143	Bragg x JS 335
G52	9–143	Multiparent cross
G53	12–31	JS 20–38 × JS 335
G54	NRC 128	JS 97–52 x (EC 389,148 × PS 1042)
G55	14–113	Bragg x JS 335
G56	12–63	JS 20–38 × JS 335
G57	6A-58–5	JS 335 × EC 538,828
G58	2A-24–1	Type 49 × EC 538,828
G59	12–71	JS 20–38 × JS 335
G60	7A-123–2-1	JS 335 × EC 538,828
G61	13–119	JS 97–52 × JS 335
G62	6A-58–2	JS 335 × EC 538,828
G63	13–41	JS 335 × Gaurav
G64	13–35	JS 335 × Gaurav
G65	NRC37 BC3F4	NRC 37 × *G. soja*
G66	226D	JS 335 × *G. soja*
G67	162 D	JS 335 × *G. soja*
G68	122 L	JS 335 × *G. soja*
C7	JS 20–69	JS 97–52 X SL 710

## Results

### Genetic variability of quantitative traits

**Figure 1:**
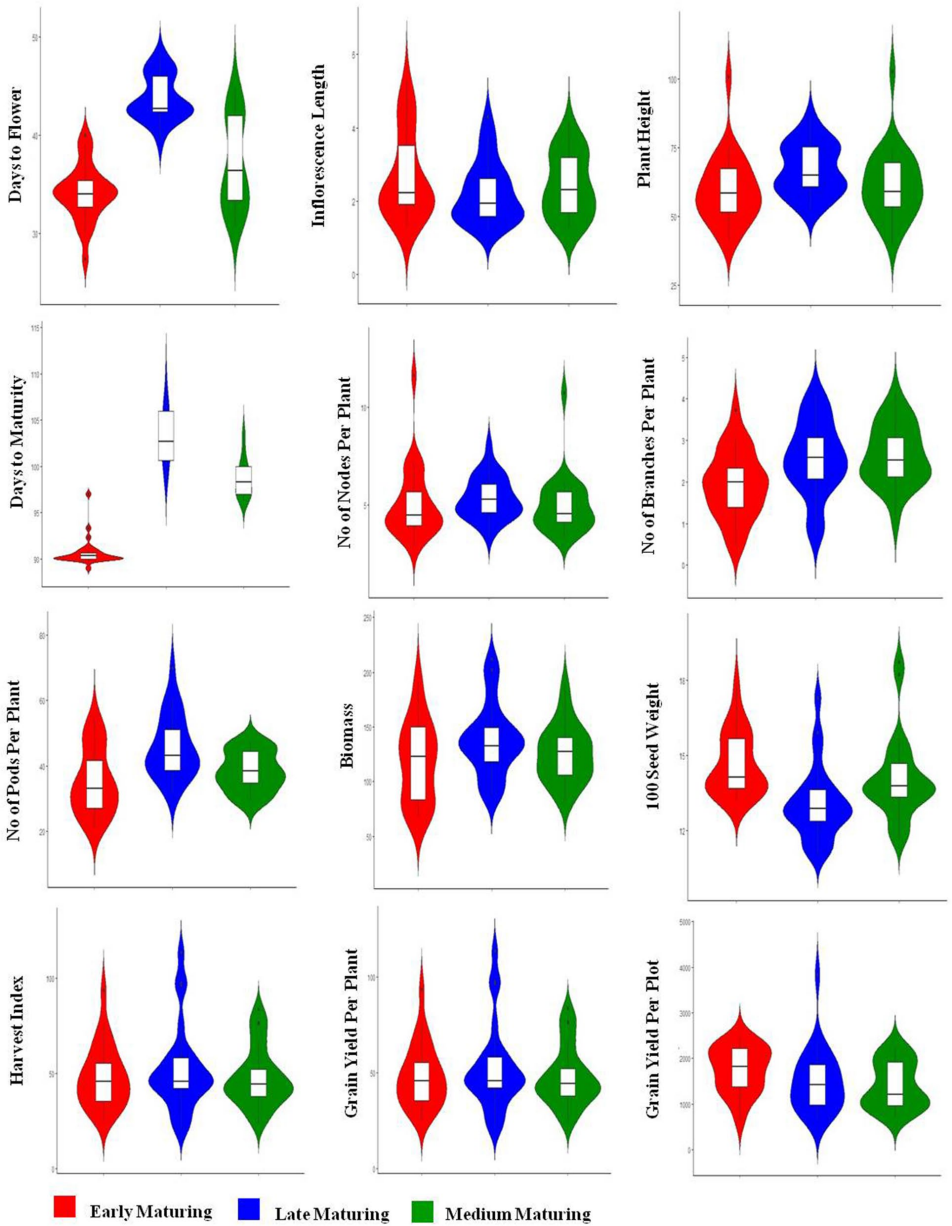
Depiction of genetic variation through violin plots for different quantitative traits in early, medium and late maturing breeding lines.

**Figure 2:**
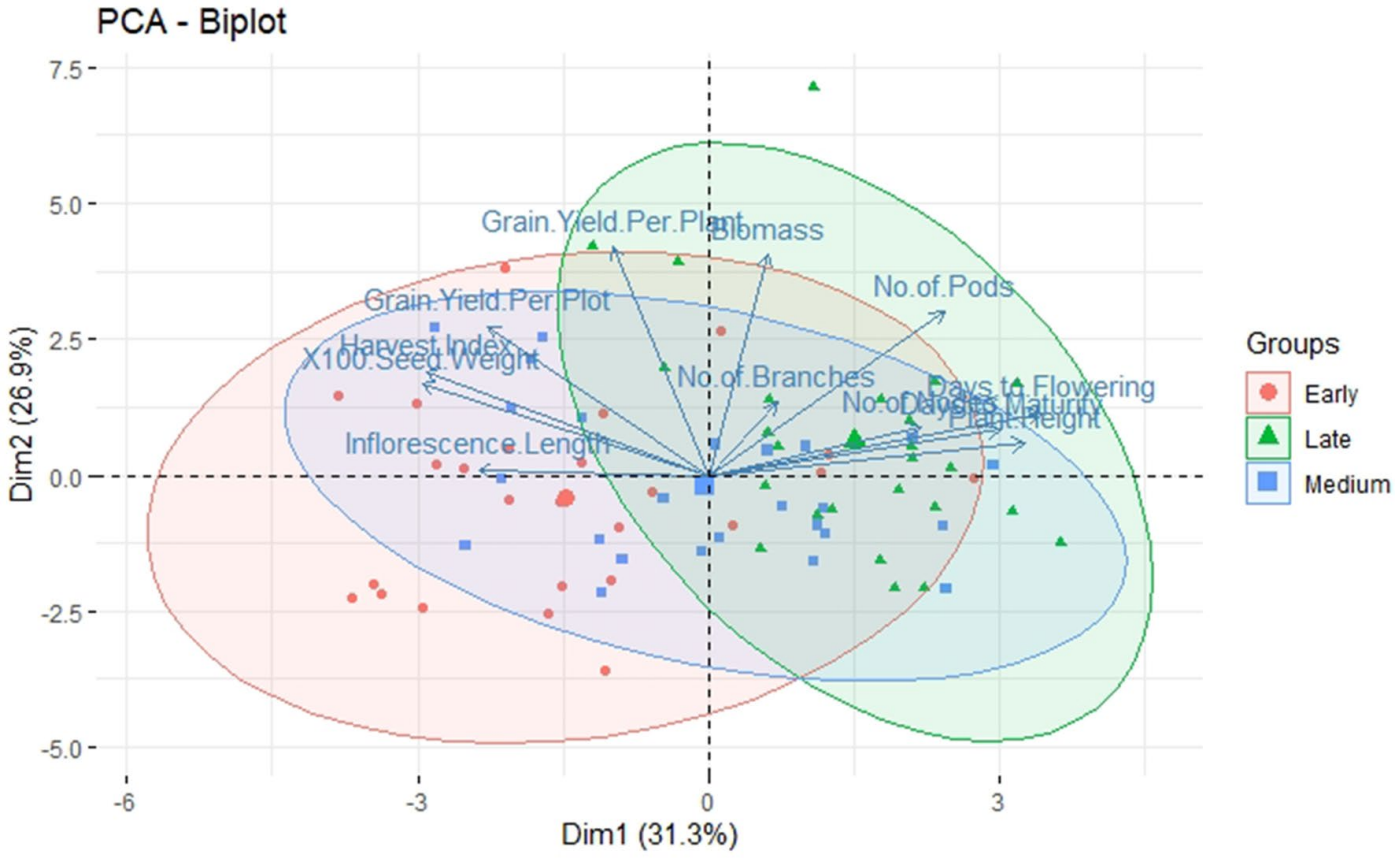
PCA for different quantitative traits in three different maturity groups (early, medium and late maturing genotypes).

Significant genotypic difference (*p* < 0.05) was observed for traits under study within individual blocks viz., early, medium and late maturity blocks except for days to maturity in early block (Tables S1–S12). Pairwise comparison of the genotypes within the three groups was analyzed through LSD test (*P* < 0.05). In the early maturity (block 1), with respect to grain yield/ plant, entry G21 (8–101–3), (93.9 g) was significantly superior to both the check varieties JS 20–34 and JS 95–60 whereas, G20 (6A–34–11) yielded (2470 g/plot) on par with check variety JS 20–34 (2344 g/plot). In block 2, G42 (6A–18–3–1) entry produced yield of 2230 kg/plot which is significantly higher than three checks JS 93–05, NRC 86 and JS 20–29. Similarly, in block 3, G54 (NRC 128) yielded 3833 kg/plot which is significantly higher than the rest of the tested entries across the block. However, as far as yield/plant is concerned it produced on par with two tested entries G 57 and G 62 (Table 2). As observed from violin plots (Fig. 1) and PCA (Fig. 2), overall, inflorescence length was highest in medium maturing group followed by early and late maturing groups. No of nodes per plant, No of branches per plant, No of pods per plant and biomass was recorded highest in late maturing group followed by medium and early maturing groups. Traits like 100 seed weight, harvest index, grain yield per plant and grain yield per plot were highest in case of early maturing group followed by late and medium maturing groups. Variability among the other traits across the blocks was shown in Table S2. Days to flowering was recorded in the range of 27–48 days. IC 15,089 an indigenous germplasm accessions flowered in only 27 days followed by entry (13–2) derived from NRC 86 × MACS 330 (30 days). It matured earlier (89 days) when compared to the other entries, whereas JS 97–52 took 48 and 100 days to flower and mature respectively. 100 seed weight was found highest (18.72 g) in G38 (6A–33–1–2) followed by G32 (8–94–3) and several entries exceeded the checks for trait 100 seed weight. The inflorescence length was found highest (5.25 cm) in G2 (6A–47–1) followed by G22 (6A–47–4) (4.76 cm). Plant height was also showed wider range (39.2–102.8 cm) and highest plant height (102.8 cm) was recorded in the genotype G26 (13–100) followed by G14 (100.8 cm) (12–16) and JS 97–52(86.27 cm).Biomass per plant (g) was showed66.00–211.67 g range and highest was recorded in the line G54 (NRC 128). (Tables S13 and S14). Narrow differences between PCV and GCV indicated lesser influence of the environment for all the twelve traits. Number of branches per plant (44.98/32.16), yield/plant (43.05/33.72) and yield/plot (40.69/36.46) were recorded higher PCV and GCV than days to flower (14.00/13.62), days to maturity (6.19/5.65), 100 seed weight (13.83/10.02), plant height (21.41/17.92) and harvest index (25.95/11.93) whereas, higher heritability was found for days to flower (0.94), plot yield (0.80), inflorescence length (0.74) and plant height (0.70) than the traits viz., harvest index (0.21), no. of branches (0.48) and 100 seed weight (0.52). Similarly, highest genetic advancement was recorded for plot yield (1042.62) and lowest for the trait inflorescence length (1.65) (Table S13).

**Table 2:** Pair wise comparison of the advanced breeding lines within early, medium and late maturity groups.

Genotype	Block I (early maturity)		Genotype	Block II (medium maturity)		Genotype	Block III (late maturity))	
	Yield/plot (g)	Yield/plant (g)		Yield/plot (g)	Yield/ plant (g)		Yield/plot (g)	Yield/ plant (g)
C1	2344.3^ab^	31.2^jk^	C3	1087.6f.^–i^	40.4^e–i^	C6	1555.3^d–g^	51.5^c–f^
C2	1381.6^e–i^	35.6^ g–k^	C4	870.6^ g–j^	42.2^e–i^	C7	1867.7^cde^	46.2^c–g^
G1	2212.3^a–c^	38.2f.^–k^	C5	1138.3^e–h^	40.6^e–i^	G46	1780.7^cdef^	45.9^c–g^
G10	1284.3f.^–i^	43.5^e–k^	G24	1647.0^ cd^	48.1^d–h^	G47	2155.7^bc^	52.0^c–e^
G11	1395.6^e–i^	48.7^c–j^	G25	967.0f.^–j^	52.2^c–g^	G48	1005.7^i–l^	42.2^c–h^
G12	2175.3^a–c^	58.2 ^b–f^	G26	904.0f.^–j^	45.3^d–i^	G49	1141.0^ h–k^	45.23^c–g^
G13	868.6^ij^	39.3f.^–k^	G27	2194.0^a^	67.9 ^a–d^	G50	1476.6^e–h^	59.73^bc^
G14	1224.6^ g–i^	44.8^d–k^	G28	1210.3^e–g^	41.7^e–i^	G51	1906.0^ cd^	58.43^b–d^
G15	1300.3f.^–i^	54.4^c–h^	G29	1111.3f.^–i^	46.6 ^d–i^	G52	619.0^ l^	37.57^d–h^
G16	2246.6^a–c^	49.3^c–j^	G30	1230.0^ef^	38.1f.^–i^	G53	1295.0^ g–i^	48.50^c–g^
G17	1443.6^d–i^	32.3^i–k^	G31	2191.0^a^	76.2^a–c^	G54	3833.3^a^	113.58^a^
G18	607.6^j^	25.0^ k^	G32	1779.3^b–d^	76.8^ab^	G55	1175.6^ g–j^	45.30^c–g^
G19	1683.6^c–h^	65.0^b–e^	G33	1915.0^a–c^	52.2^c–g^	G56	974.0^i–l^	45.50^c–g^
G2	2243.3^a–c^	67.8^bc^	G34	1477.3^de^	38.03f.^–i^	G57	2359.3^b^	96.7^a^
G20	2470.3^a^	55.5^b–g^	G35	1058.0f.^–j^	27.3^hi^	G58	1424.6f.^–h^	36.1^e–h^
G21	2236.0^a–c^	93.93^a^	G36	939.3f.^–j^	32.6 g^–i^	G59	1562.3^d–g^	55.6^b–e^
G22	2054.3^a–c^	66.0^b–d^	G37	2094.6 ^ab^	52.0^c–g^	G60	2165.6^bc^	75.4^b^
G23	1943.3^a–e^	45.8^d–k^	G38	1942.3^abc^	83.9^a^	G61	1713.3^d–f^	61.4^bc^
G3	1826.3^b–f^	76.4^ab^	G39	706.0^j^	23.3^i^	G62	1882.0^ cd^	97.6^a^
G4	1721.3^c–g^	33.60^ h–k^	G40	1931.0^abc^	31.0^ g–i^	G63	866.33^j–l^	44.1^c–g^
G5	1924.0^a–e^	46.6^c–k^	G41	759.00^ij^	34.3f.^–i^	G64	985.0^i–l^	45.4^c–g^
G6	2007.6^a–d^	34.8^ g–k^	G42	2230.0^a^	64.8^a–e^	G65	970.0^i–l^	30.6f.^–h^
G7	2416.6^ab^	53.0^c–i^	G43	1212.3^efg^	44.4^d–i^	G66	1210.0^ g–j^	28.8^gh^
G8	1101.0^ h–j^	40.6f.^–k^	G44	1636.6^ cd^	57.5^b–f^	G67	760.3^kl^	22.0^ h^
G9	1653.6^c–h^	26.9^ k^	G45	841.0^hij^	41.7^e–i^	G68	637.3^ l^	22.0^ h^

Pairwise comparison using LSD (*p* < 0.05).

### Correlation analysis

**Figure 3:**
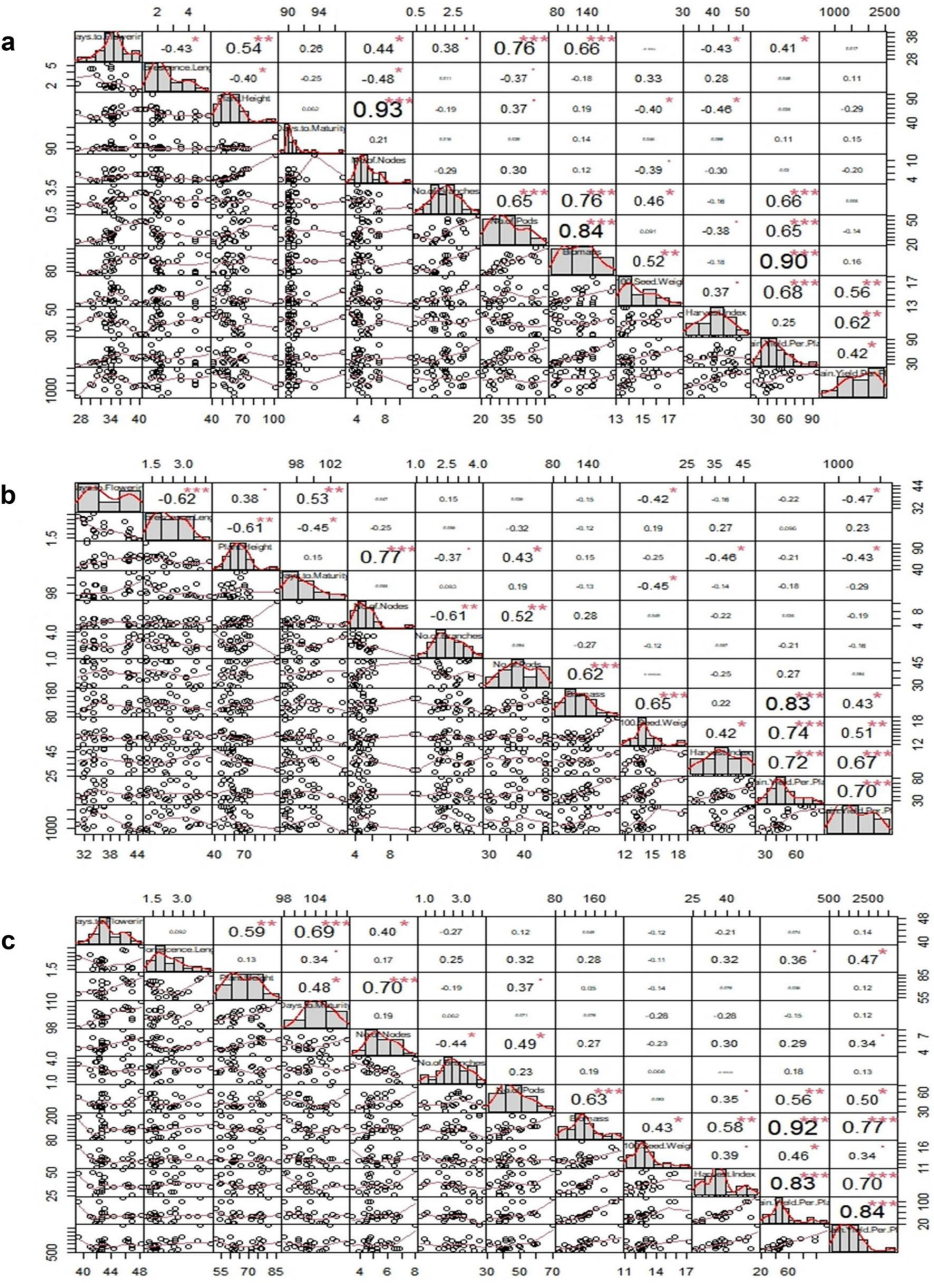
Correlation analysis in early (**a**), medium (**b**), late (**c**) maturing advanced breeding lines of soybean. (Generated using R package “PerformanceAnalytics version 2.0.4 URL https://github.com/braverock/PerformanceAnalytics).

Correlation of yield with other traits in early maturing breeding lines revealed that grain yield per plant had significant positive correlation with 100 seed weight (0.68***), biomass (0.90***), pods per plant (0.65***), branches per plant (0.66***) and days to flowering (0.41*). In the medium maturing, yield per plant was significantly associated with harvest index (0.72***), 100 seed weight (0.74***) and biomass (0.83) but found non-significant negative association with days to flowering (-0.22), plant height (− 0.21), days to maturity (− 0.18) and branches per plant (− 0.21). In case of late maturing breeding lines, yield per plant was significantly associated with harvest index (0.83***), biomass per plant (0.92***), pods per plant (0.56**) and non-significant negative association was found with days to maturity (− 0.15). Inflorescence length was significantly positive association with grain yield per plot (0.47*) (Fig. 3).

### Cluster analysis

In the present study, days to flowering (52.25%) exhibited greater variation and contribution to diversity among genotypes followed by days to maturity (10.38%) and plot yield (9.23%). Yield per plant (1.15%), harvest index (0.22%) and branches per plant (1.73%) contributed comparatively less to the total diversity (Table 3). Seventy-five genotypes including seven checks grouped into five clusters based on D^2^ values using the Tocher’s method. The distribution of genotypes into various clusters is depicted in Fig. S1 & Table 4. Out of the five clusters, cluster I was the largest comprising of 57 genotypes followed by cluster II with 14 genotypes. Clusters III and V represented by one genotype each and two genotypes were presented in cluster IV. The average intra and inter cluster D^2^ values can be computed from the cluster diagram where the statistical distances among the 75 genotypes were exhibited (Table S15). Intra cluster D2 values ranged from zero to 6.79 with maximum distance in cluster 1 (8.04), followed by cluster IV (6.17). From the inter cluster D^2^ values of the five clusters, highest divergence was noticed between cluster II and V (19.76) while the lowest was noticed between cluster III and V (10.17). The cluster means for each of 12 characters (Table S16) indicated that the cluster mean for days to flowering was highest in cluster III (48.33) and the lowest in cluster II (31.67) and similar trend was noticed with days to maturity with respective clusters. 100-seed weight was highest in cluster II (14.70 g) and lowest in cluster III (12.37 g). Cluster V recorded the highest plot grain yield (3833.33 g) and the lowest was in cluster IV (1064.33). Cluster III was characterized by longest inflorescence (4.29 cm) while the shortest was recorded in cluster IV (1.49 cm). The number of pods per plant was highest in cluster V (70.0) and the lowest number was noticed in cluster II (29.77). It was observed that cluster V had many of the desirable means for several characters and with respect to contribution to the genetic diversity.

**Table 3:** Relative contribution of twelve different characters to total genetic diversity.

Source	Times ranked1st	Contribution (%)
1 Days to flower	1450	52.25
2 Inflorescence length (cm)	225	8.11
3 Plant height (cm)	116	4.18
4 No. of nodes/Plant	83	2.99
5 No. of branches/Plant	48	1.73
6 No. of pods/Plant	62	2.23
7 biomass (gm)	167	6.02
8 plot yield (g)	256	9.23
9 yield/plant (gm)	32	1.15
10 Days to maturity	288	10.38
11 100^–^Seed weight (gm)	42	1.51
12 Harvest Index (%)	6	0.22

**Table 4:** Grouping of 75 advanced breeding lines into five different clusters based on twelve quantitatively contributing traits by D2 analysis.

Cluster	No. of lines	Genotype
Cluster I	57	G1, G3, G5, G7, G10, G11, G12, G13, G15, G16, G19, G21, G20, G23, C3, G24, G25, C4, G27, G29, G31, G32, G33, G34, G36, G37, G39, G40, G41, G42, G43, G44, G45, C5, G46, G47, G48, G49, G50, G51, G52, G53, G55, G56, G57, G58, G59, G60, G61, G62, G63, G64, G65, G66, G67, G68, C7
Cluster II	14	G2, G4, G6, C1, C2, G8, G9, G17, G18, G22, G28, G30, G35, G38
Cluster III	01	C6
Cluster IV	02	G14, G26
Cluster V	01	G54

### Selection of genotypes based on MGIDI index and genetic gain

**Figure 4:**
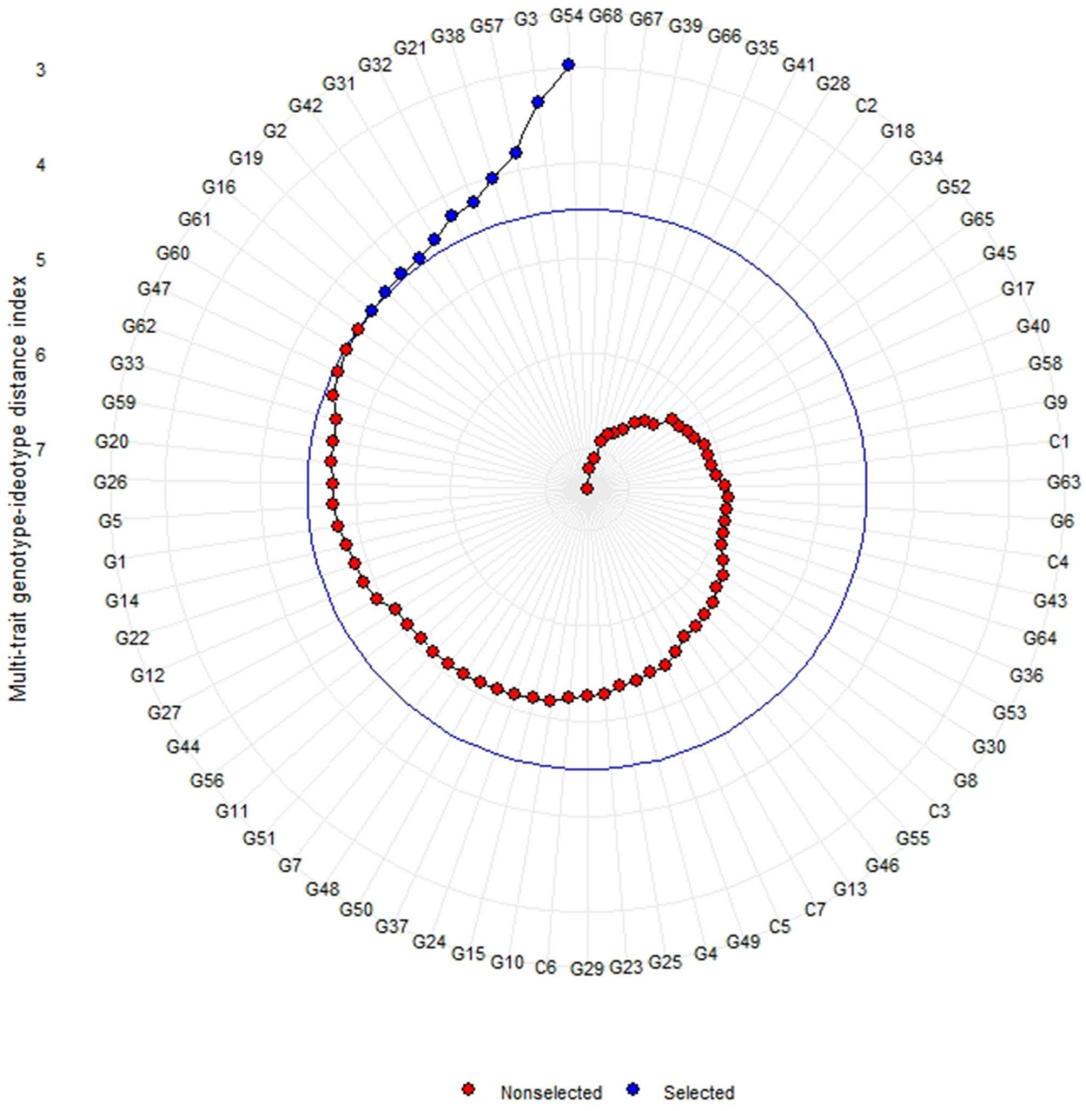
MGIDI index and selection of superior genotypes (Generated using R Package ‘metan’ version 1.15.0 URL https://github.com/TiagoOlivoto/metan).

Based on multi-trait genotype-ideotype distance index (MGIDI), eleven genotypes viz., G54 (NRC 128), G3 (3A–60–6), G57 (6A–38–5), G38 (6A–33–1–2), G21 (8–101–3), G32 (8–94–3), G31 (6A–34–25), G42 (6A–18–3–1), G57 (6A–38–5), G2 (6A–47–1), G19 (8–98–1) and G16 (3A–44–1–1) were selected as superior to the others (Fig. 4). G61 (13–119) was very close to the cut point (blue line that indicates genotypes selected according to the selection pressure). The genetic gain results based on MGIDI revealed that MGIDI was the most efficient index to select genotypes with desired characteristics. The only trait with negative selection gain (− 1.91%) was observed with plant height. The highest genetic gain reported for plot yield (633) followed by biomass (33.8), yield per plant (24) and pods per plant (4.57) (Table 5).

**Table 5:** Genetic gain for different traits based on MGIDI Index.

Factor	Traits	Objective	Genetic gain
FA1	Days to flowering	Decrease	− 1.97
FA1	No. of branches/ plant	Increase	0.139
FA1	No. of pods/plant	Increase	4.57
FA1	Days to maturity	Decrease	− 2.20
FA2	Biomass (g)	Increase	33.8
FA2	Plot grain yield (g)	Increase	633
FA2	Yield/plant (g)	Increase	24
FA2	100 seed weight (g)	Increase	1.38
FA2	Harvest index (%)	Increase	3.49
FA3	Inflorescence length (cm)	Increase	0.471
FA3	Plant height (cm)	Increase	− 1.91
FA3	No. of nodes/plant	Increase	0.227

### Evaluation for waterlogging tolerance

Evaluation of three genotypes for waterlogging tolerance at vegetative stage revealed that NRC 128 performed on par with tolerant check JS 97–52 with respect to two important waterlogging tolerance traits viz., percentage reduction of chlorophyll content and seed yield. Percentage reduction in root nodule dry weight, foliar damage score and plant survival rate were also relatively comparable with JS 97–52. Waterlogging tolerance coefficient was 77.65 for NRC 128 whereas it was 72.11 for JS 97–52, showing better performance of NRC 128 (Table 6). The susceptible check performed very poor when compared with JS 97–52 and NRC 128. Same genotypes were evaluated during reproductive stages, out of three, NRC 128 was found to perform on par (WLTI = 0.91) with JS 97–52 (WLTI = 0.90) in terms of reduction in seed yield per plant during waterlogging stress. In addition, it performed superior to best check in terms of 100 seed weight under stress conditions. Susceptible check, JS 90–41 found inferior in all the traits recorded when compared to tolerant check and NRC 128 (Table 7).

**Table 6:** Evaluation of NRC 128 for waterlogging tolerance at vegetative stage (V_2_ – V_3_) under controlled conditions.

Genotype	Foliar damage score	Plant survival rate (%)	Stem elongation rate (%)	% reduction in root nodules dry weight per plant	% reduction in SCMR (SPAD chlorophyll meter readings)	% reduction in seed yield per plant	waterlogging tolerance coefficient
NRC 128	2	95.59	103.57	6.45	14.92	18.77	77.65
JS 97–52 (Tolerant Check)	1.67	97.37	118.81	5.92	15.39	25.96	72.11
JS 90–41 (Susceptible Check)	5.21	75.19	85.00	52.30	26.85	45.51	40.97

**Table 7:** Evaluation of NRC 128 for waterlogging tolerance at reproductive (R1) stage under controlled conditions.

Soybean genotypes	% reduction in total chlorophyll content	% reduction in no. of pods per plant	% reduction in 100 seed weight	% reduction in seed yield per plant	waterlogging tolerance index
NRC 128	29.49	27.47	1.46	9.49	0.91
JS 97–52 (Tolerant Check)	22.78	11.33	14.51	9.64	0.90
JS 90–41 (Susceptible Check)	46.28	32.59	22.38	38.28	0.62

### G × E analysis

**Figure 5:**
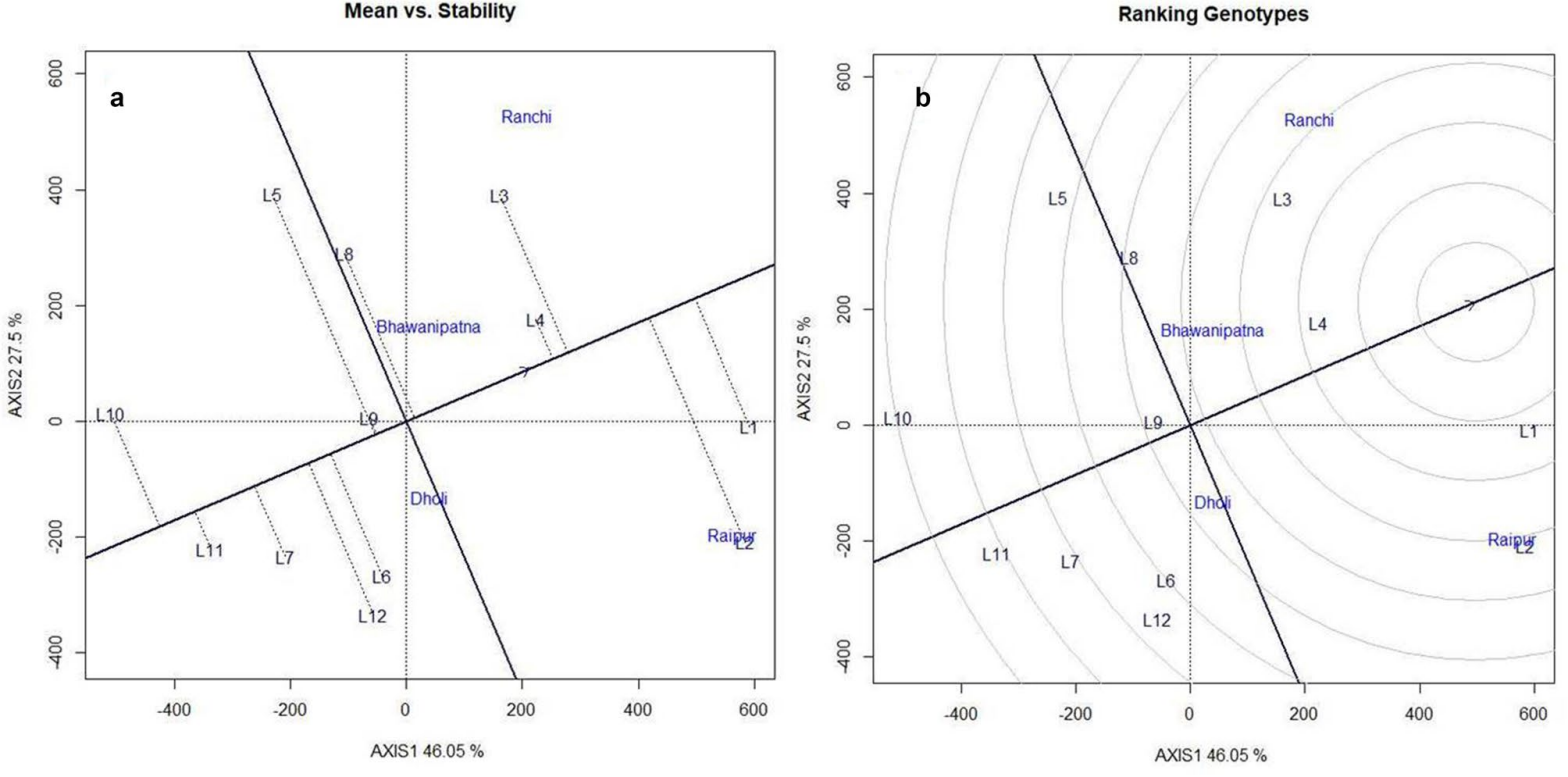
GGE Biplot analysis of genotypes evaluated in Eastern Zone: (**a**) Mean vs Stability analysis, (**b**)- Ranking of genotypes based on ideal genotype: L1-RAC 11–07, L2-NRC 128, L3-AMS 2014–1, L4- NRC 136, L5- MACS 1493, L6- RSC 11–03, L7-NRCSL 1, L8-NRC 132, L9-NRC 137, L10-JS 335, L11-RKS 18 and L12- JS 97–52.

**Figure 6:**
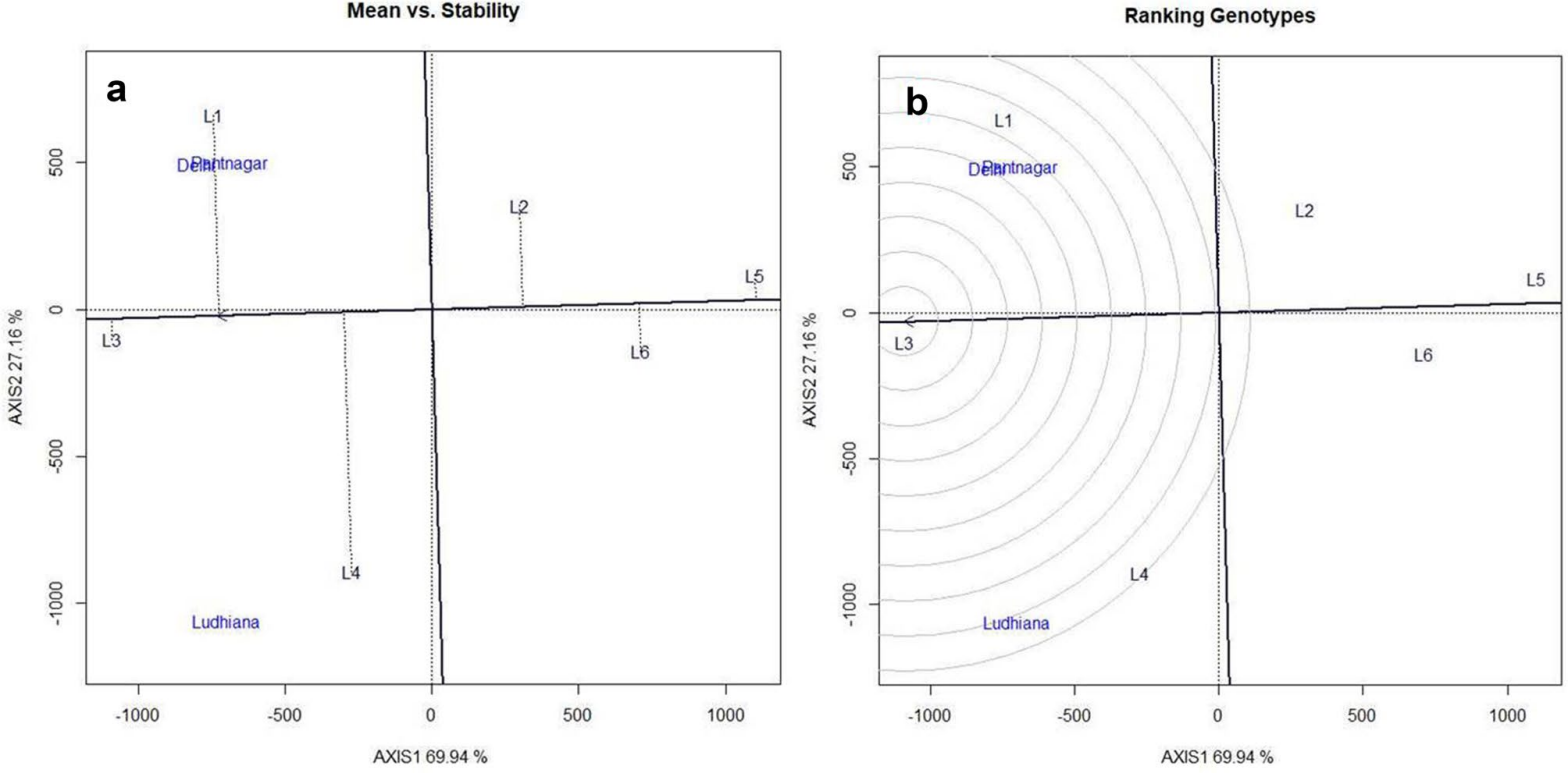
GGE Biplot analysis of genotypes evaluated in Northern Plain Zone: (**a**) Mean vs Stability analysis, (**b**) Ranking of genotypes based on ideal genotype: L1-PS 1613, L2-PS 1611, L3- NRC 128, L4- PS 1347, L5-Pusa 97–12 and L6-SL 958.

**Figure 7:**
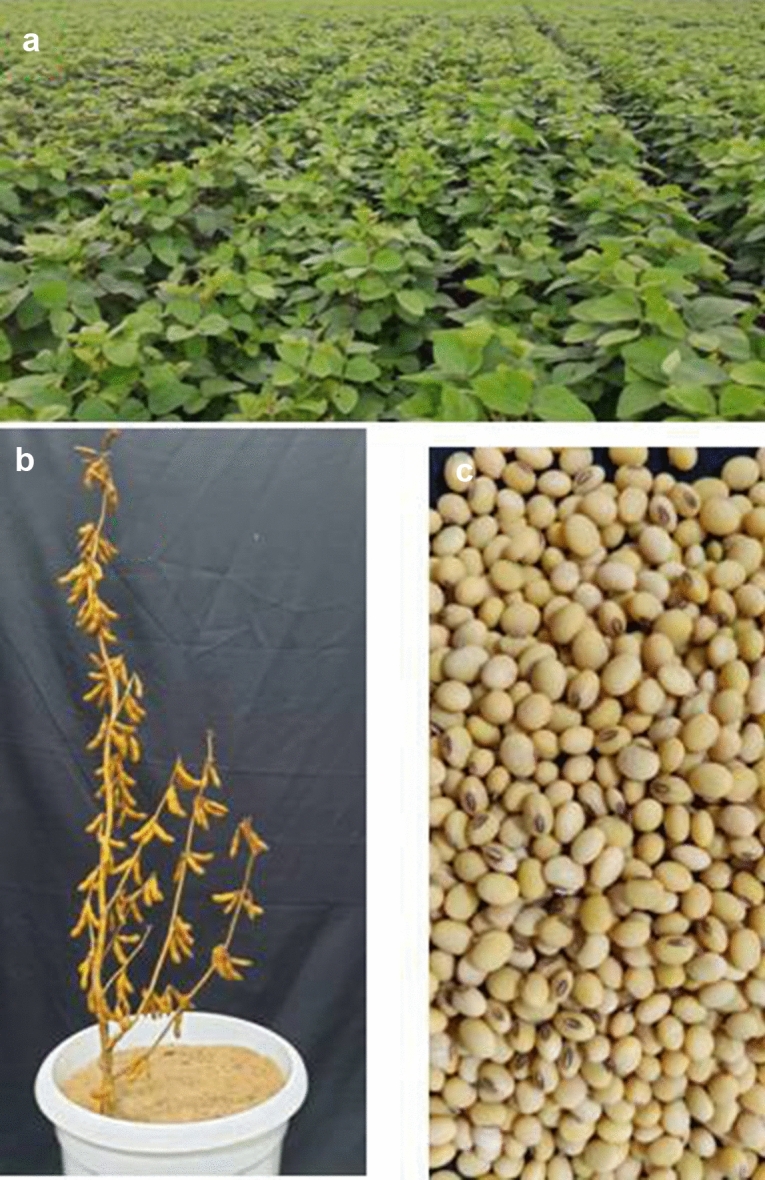
NRC 128 newly released waterlogging tolerant variety (**a**) Crop at vegetative sage (**b**) Single plant (**c**) Seed color and shape.

For testing the superiority of promising genotype NRC 128 (L2),eleven other promising genotypes (RSC 11–07 (L1), AMS 2014–1 (L3), NRC 136 (L4), MACS 1493 (L5), RSC 11–03 (L6), NRCSL 1 (L7), NRC 132 (L8), NRC 137 (L9), JS 335 (L10), RKS 18 (L11) and JS 97–52 (L12)were evaluated at four locations Raipur, Dholi, Bhawanipatna and Ranchi (Eastern zone). Through mean vs stability analysis, RSC 11–07 was found to be high yielding (1755.50 kg/ha) followed by NRC 128that produced 1720 kg / ha of grain yield which was9.76% higher yield than the best check JS 97–52(1552 kg/ha) (Fig. 5a). The 100 seed weight of the NRC 128 (13.2 g) was also higher than that of all the check entries (Table 8). On the other hand, RSC 11–07 was found to be near ideal genotype followed by NRC 136, AMS 2014–1 and NRC 128when mean performance and stability were considered simultaneously (Fig. 5b). Similarly, in Northern plain Zone, NRC 128 (L3) was evaluated along with five other promising entries viz., PS 1613 (L1), PS 1611 (L2), PS 1347 (L4), Pusa 97–12 (L5) and SL 958 (L6). It ranked first in terms of mean performance and stability by yielding 2242 kg / ha which was 20.6% higher than best check PS 1347 (1782 kg/ha) (Fig. 6a). Further, NRC 128 was ranked first with respect to ideal genotype (Fig. 6b). The mean multi-location data for grain yield (kg/ha) for NPZ and EZ has been presented in Tables S17 & S18 respectively. Pooled ANOVA for genotypes evaluated across two agro-climatic Zones were presented in Table S19. Phenotype of NRC 128 has been depicted in the Fig. 7.

**Table 8:** Mean performance and superiority for agronomic traits of NRC 128 over best checks in Eastern Zone and Northern plain Zone.

Zone/Locations	Varieties	Mean yield (kg/ha)	Maturity (Days)	100 SW (gm)	% yield increase over best check
Eastern Zone (Bhawanipatana, Dholi, Raipur, Ranchi)	NRC 128	1720	106	13.2	9.76
	JS 335(Check)	1394	105	10.46	
	RKS 18(Check)	1444	107	11.47	
	JS 97–52(Check)	1552	110	10.39	
Northern plain Zone (Delhi, Ludhiana, Pantnagar)	NRC 128	2242	117	9.92	20.51
	Pusa 97–12(Check)	983	121	8.01	
	PS 1347(Check)	1782	123	8.94	
	SL 958(Check)	1200	126	8.77

### Offseason seed multiplication

For the seed requirement of the farmers, 477 kg (4.77 quintal) nucleus seed of NRC 128 was sown at Belagavi, Karnataka during February- May 2021. The variety was sown in area of 7.2 hectare and produced 7020 kg (70.20 quintal) seed with productivity of 975 kg per hectare.

## Discussion

With the presence of narrow genetic base in soybean, use of diverse parents and development of large F_2_ population plays a vital role in development of high yielding varieties. Therefore, employing exotic germplasm accessions in hybridization program will helps in broadening the genetic base. Yield potential is built-up by progressive assembling of productivity genes as against quality, resistance to biotic and abiotic stresses^21^. In India, mega soybean varieties like Gaurav (JS 72–44), JS 335, JS 93–05 and JS 95–60 were bred through conventional breeding and aided in enhanced soybean production^21^. The annual genetic gain during 1969 to 1993 in seed yield of Indian soybean varieties has been about 22 kg/ha^22^. Similar gain trend was also seen in other reports^23^.

For increasing the yield per se in soybean crop, conventional breeding must be reoriented with use of discreetly chosen parents and pre-bred diverse material in the crosses, sizeable F_2_ populations and three-way crosses, multiparent crosses and combination breeding^21^. An experiment was carried out in group balanced block design (GBBD) which is very efficient compared to regular RBD design. The GBBD design helps in reduction of experimental error by making blocks based on the maturity of the genotypes and treatments are compared with higher degree of the precision^24^. The presented study evaluated early, medium, and late maturing soybean advanced breeding lines for yield and attributing traits. The advanced breeding materials were derived from diverse crosses, including pre breeding material derived from wild type *G. soja* andmultiparent crosses. Two germplasm accessions EC 572,109 and IC 15,089 (triple mutant for e1, e3, e3) carrying early maturity alleles were also evaluated for yield and associated traits and both matured in 89–90 days^25^. One of the advanced breeding line 13–2 derived from MACS 330 × NRC 86 where, MACS 330 (IC538550) is a source for photoperiod insensitivity and recessive alleles e2, e3-tr, and extra early maturity (< 85 days)^26^. The early maturing breeding lines evaluated in block 1, particularly those derived from crosses involving one of the parent as EC 538,828 was found to have higher 100 seed weight than other tested entries in block 2 and block 3. EC 533,828 is bold seeded genotype along with tolerance to drought and terminal heat stress^27^. Breeding for early maturity is much needed to fulfill the demand of the farmers in the Central India. In this region, early maturity soybean is primary requirement for soybean-potato-wheat/soybean-wheat cropping system. In the present study, few early maturing breeding lines 6A-34–11 (NRC 146) (2470 kg/plot), 6A-34–6 (2416 kg/plot) and 3A-44–1-1 (2246 kg/plot) yielded on par with best early maturing check JS 20–34 (2344.33 kg/plot).The genotype NRC 146 was reported as heat tolerant^28^.

The response to selection in any crop improvement program depends on the degree of genetic variability and heritability^29^. High degree of genetic parameters such as heritability, variance, genetic advance, and genetic gain for the important traits like grain yield, biomass and others has been noticed in the current study. Correlation analysis in the present study revealed that improvement of higher yield is possible through selection of attributing traits such as biomass, harvest index, plant height, number of branches, number of pods, which was in accordance with previous reports^30,31^. Soybean varieties with ideal inflorescence architecture could help in producing more yield potential. As an important and complex trait, inflorescence length (IL) of soybean significantly affected seed yields^32,33^. In the present study longest inflorescence was found in G2 (6A–47–1) genotype (5.25 cm) and it has also positive and significantly correlated with yield per plot (0.47*). Cluster analysis helps in identification of distinct and diverse genotypes for the hybridization program to develop breeding material with broader genetic base. In the present study it grouped the accessions into five clusters with cluster I comprising of 57 accessions indicating close relatedness of the accessions and crossing among these accessions may yield less genetic gain. Such reports on grouping of genotypes were done by other workers^34,35^. Clusters III and V had only one breeding line each indicating a high degree of heterogeneity, and these may be directly utilized as parents in hybridization programs to combine desirable characters. Similarly, hybridization between lines belonging to different clusters especially cluster II and V certainly is rewarding in generating diverse breeding material.

An efficient multivariate selection index, MGIDI^2^ was used to select genotypes nearer to ideotype. Based on this index, eleven genotypes were selected as superior to other tested entries and out of eleven, genotype G54 (NRC 128) is found to be ranked first in terms of ideotype. The genetic gain was positive for the traits under consideration except with plant height. Plant height had found negative gain may be due to its negative association with yield attributing traits viz., branches per plant, yield per plot, harvest index and 100 seed weight (Fig. S2). NRC 128 genotypes is derived from JS 97–52 x (EC 389,148 × PS 1042) and one of its parents, JS 97–52 is climate smart genotype having resistance for major disease of soybean such as charcoal rot and yellow mosaic disease, and tolerance to abiotic stresses like drought, heat, and waterlogging stresses^36–40^. JS 97–52 possesses 100-seed weight of 8–9 gm, whereas in the NRC 128, 100 seed weight trait has been improved to 13.3 g and it was also least affected by waterlogging stress. The importance of GGE is demonstrated in number of other crops for yield and other agronomic traits^41–46^ to understand G x E interaction pattern and to select stable and superior genotypes. Based on GGE biplots RSC 11–07 was found to be near-ideal genotype in Eastern Zone, while NRC 128 was found to be near-ideal genotype in Northern Plain Zone.

Substantial yield reductions in soybean have been observed when excessive soil water occurs during both vegetative and reproductive stages of the plant^47–53^. The most effective and economic approach to decrease yield loss is by developing waterlogging tolerant soybean cultivars^17^. Screening of genotypes at reproductive stages for identification of key genes and additional genetic resources for waterlogging tolerance was emphasized^54^. In the present study, NRC 128 exhibited waterlogging tolerant at both vegetative and reproductive stages. However, this genotype was found inferior for some traits i.e. stem elongation traits, pods per plant etc. under waterlogging stress, this type of observations were also reported by researchers in earlier study^55^. The present study observed that good grain filling during waterlogging stress at reproductive stage in NRC 128; therefore, it may be one of the candidate donor parents for development of varieties for the ecologies where more rainfall occurs near harvesting stage. Availability of quality seed is one of the primary requirements of the farmers to achieve more production. A total of 70.20 quintal of NRC 128 seed was produced for farmer’s requirement. The variety as produced 975 kg per ha yield during off season which is considerably high.

## Conclusion

Evaluation of large number of advanced breeding lines identified several promising lines for early maturity, bold seed, waterlogging tolerance and higher yield. The group balanced block design and MGIDI index used in the current study were found efficient and aided in identification of NRC 128, as high yielding and near-ideotype. Further, NRC 128 identified as first waterlogging tolerant variety for northern plain zone and especially for eastern zone of India where waterlogging situations occurs due to prolonged monsoon rains. Based on its superiority over yield and waterlogging tolerance, it has been released and notified (S.O. 500(E) 29.01.2021) by central varietal and release committee of Government of India for commercial cultivation in the states of Punjab, Uttar Pradesh (except Bundelkhand region of Uttar Pradesh), Delhi, West Bengal, Bihar, Jharkhand, Chhattisgarh, and Orissa.

## Material and methods

### Breeding trial

Sixty-eight advanced breeding lines derived from different crosses were evaluated for yield and attributing traits at ICAR-Indian Institute of Soybean Research, Indore, India. Group Balanced Block Design was followed for block-wise evaluation of early (up to 90 days), medium (up to 100 days) and late maturing (up to 110 days) genotypes. Genotypes were grouped into three blocks each with 25 genotypes including seven checks (early maturing checks –C1 (JS 20–34) and C2 (JS 95–60); medium maturing checks –C3 (JS 93–05), C4 (JS 20–29) and C5(NRC 86); late maturing checks –C6 (JS 20–69) and C7 (JS 97–52). The complete list of genotypes and their pedigree were presented in Table 1. Each genotype was evaluated in three replicates and each replication was sown in a plot of size 13.5 m^2^. Data on twelve quantitative traits viz., Days to flowering, Days to maturity, No. of branches, No. of nodes, Plant height (cm), inflorescence length (cm), No. of pods per plant, biomass per plant (g), 100 seed weight (g), harvest index (%), grain yield per plot (g)and grain yield per plant (g) were recorded as per standard procedure (IBPGR 1984). Grain yield per plant was based on average yield (g) of five randomly selected plants. Recommended crop production package of practices has been followed throughout the experiment to reach maximum yield potential of the crop^56^. The methodology andprotocol used in the present study are in accordance with relevant institutional, national, and international guidelines and legislation.

### Evaluation for waterlogging tolerance

Three genotypes viz., NRC 128, JS 97–52, and JS 90–41 were evaluated for waterlogging tolerance at early vegetative and reproductive growth stages at ICAR-Indian institute of Soybean Research, Indore. As per our previous records NRC 128 was found promising and it has derived from the waterlogging tolerant variety JS 97–52; therefore, it was evaluated along with two check varieties for waterlogging tolerance under controlled conditions. All three genotypes were sown in three rows of one meter each and observations were recorded on every individual plant. For vegetative stages waterlogging tolerance, waterlogging stress was imposed during V_2_-V_3_growth stages for 10 days by saturating the soil up to 10 cm above the soil surface in stress field plot while counter control field plot was maintained with normal irrigated condition using standard protocols^57,58^. Foliar damage score (FDS; 1–9 scale based on chlorosis, necrosis and plant mortality), plant survival rate (PSR)^59^ and stem elongation rate (SER) in stressed plot were recorded. Plant survival rate was calculated as: PSR = {100 − (number of plants before stress—number of plants after stress/ number of plants before stress)} × 100. Stem elongation rate (SER) was calculated as: = (height after stress- height before stress)/ height before stress × 100. For determining the leaf chlorophyll content in both plots, five unrolled leaflets were randomly selected in each replicate using a chlorophyll meter (Konica Minolta, SPAD-502).Similarly, root nodule dry weight per plant in both plots (control and stress) was estimated as per methodology suggested^60^. After recording the yield traits and other related morpho-physiological traits in control and stress plots, percent reduction in grain yield per plant, root nodules’ weight and SCMR (SPAD chlorophyll meter readings) under waterlogged conditions in comparison to normal field conditions was estimated. Waterlogging tolerance coefficient (WTC) was calculated with formula WTC = mean value (seed yield per plant) of treatment (genotype) in stressed plot × plant survival rate/mean value (seed yield per plant) of treatment (genotype) in control plot.

Similarly, NRC 128 along with susceptible and tolerant check was evaluated for waterlogging tolerance at reproductive stage. Waterlogging stress was provided at R_1_ stage (12–15 cm of water above the soil surface) for 15 days as per methodology with slight modifications^61^. These genotypes were evaluated for yield attributes^62^ and total chlorophyll content (through Acetone DSMO method) as per methodology from control and stress plot^63^. Waterlogging tolerance was evaluated by dividing the seed yield of stressed plants by that of the control plants, to provide a waterlogging tolerance index (WTI)^64^.

### Multi-location evaluation

A total eleven genotypes along with NRC 128 was evaluated for yield in different locations of Eastern Zone (Dholi, Raipur, Ranchi, Bhavanipanta). Similarly, total of six genotypes including NRC 128 were evaluated at three locations viz., Delhi, Ludhiana and Pantnagar in North Plain Zone (of India. Multi-location trials were conducted in RBD fashion with four replicates each. Each replication is sown in a plot size of 21.6 m^2^ and the yield was converted into kg/ha. Recommended package of practiced were followed throughout the experiments^56^. Finally, nucleus seeds of NRC 128 genotype were multiplied in offseason at Belagavi, Karnataka (February-May 2021) for farmers requirement.

### Statistical analysis

For the breeding trial, Analysis of Variance (ANOVA) was calculated as per Gomez and Gomez. Violin plots for different traits were generated using “ggplot2” R package^65^. Correlation analysis has been carried out using R package “PerformanceAnalytics”^66^. Principal Component Analysis was done using R packages “devtools”^67^ and “factoextra”^68^. Cluster analysis was carried out using software “INDOSTAT”. For multi-location trials, GGE Biplot analysis was done using R package “GGEBiplotGUI”^69^. MGIDI index was calculated using R package “Metan”^70^.

## Supplementary Information

Supplementary Information.

## Data Availability

The raw data supporting the conclusions of this manuscript will be made available by the authors to any qualified researcher. All datasets used for analysis in the study are included in the manuscript and as supplementary files.
