# Supplementary material for: Breeding for higher yield, early maturity, wider adaptability and waterlogging tolerance in soybean (Glycine max L.): A case study
Source: Sci Rep. 2021 Nov 24;11:22853. doi: 10.1038/s41598-021-02064-x (PMC8613251; doi:10.1038/s41598-021-02064-x)
Supplement: Supplementary file 1 — Supplementary Information. [file 41598_2021_2064_MOESM1_ESM.docx]

**Table S1. Analysis of Variance (Group Balanced Block Design) for Days to Flowering**

|  | | | | | Tabular F |  |
| --- | --- | --- | --- | --- | --- | --- |
| Source of Variation | DF | Sum of Square | MSS | Computed F(b) | 0.05 | 0.01 |
| Replication | 2 | 4.808889 | 2.404444 |  |  |  |
| Varietal Group | 2 | 3651.209 | 1825.604 | 523.9298 | 6.94 | 18 |
| Error(a) | 4 | 13.93778 | 3.484444 |  |  |  |
| Varieties within group A | 24 | 583.2533 | 24.30222 | 16.30819 | 1.593 | 1.923 |
| Varieties within group B | 24 | 1532.667 | 63.86111 | 42.85448 | 1.593 | 1.923 |
| Varieties within group C | 24 | 383.3333 | 15.97222 | 10.71828 | 1.593 | 1.923 |
| Error(b) | 144 | 214.5867 | 1.490185 |  |  |  |
| Total | 224 | 6383.796 |  |  |  |  |
| cv(a) %=4.87 | | cv(b) %= 3.18 | | | | |

**Inference: Results indicate the significant difference among the mean of the three groups of varieties, and significant differences among the varieties in each of the three groups.**

**Table S2: Analysis of Variance (Group Balanced Block Design) for Inflorescence Length**

|  | | | | | Tabular F |  |
| --- | --- | --- | --- | --- | --- | --- |
| Source of Variation | DF | Sum of Square | MSS | Computed F(b) | 5% | 1% |
| Replication | 2 | 0.88 | 0.44 |  |  |  |
| Varietal Group | 2 | 7.37 | 3.68 | 11.83 | 6.94 | 18.00 |
| Error(a) | 4 | 1.25 | 0.31 |  |  |  |
| Varieties within group A | 24 | 99.23 | 4.13 | 14.31 | 1.593 | 1.923 |
| Varieties within group B | 24 | 54.90 | 2.29 | 7.92 | 1.593 | 1.923 |
| Varieties within group C | 24 | 50.50 | 2.10 | 7.28 | 1.593 | 1.923 |
| Error(b) | 144 | 41.59 | 0.29 |  |  |  |
| Total | 224 | 255.71 |  |  |  |  |
| cv(a) % = 22.71 | | cv(b) %= 21.87 | | | | |

**Inference: Results indicate the significant difference (@ 5%) among the mean of the three groups of varieties, and significant differences (@1%) among the varieties in each of the three groups.**

**Table S3: Analysis of Variance (Group Balanced Block Design) for Plant Height**

|  | | | | | Tabular F |  |
| --- | --- | --- | --- | --- | --- | --- |
| Source of Variation | DF | Sum of Square | MSS | Computed F(b) | 5% | 1% |
| Replication | 2 | 327.78 | 163.89 |  |  |  |
| Varietal Group | 2 | 2363.37 | 1181.68 | 9.10 | 6.94 | 18.00 |
| Error(a) | 4 | 519.32 | 129.83 |  |  |  |
| Varieties within group A | 24 | 11515.06 | 479.79 | 9.22 | 1.593 | 1.923 |
| Varieties within group B | 24 | 11920.69 | 496.70 | 9.54 | 1.593 | 1.923 |
| Varieties within group C | 24 | 6256.80 | 260.70 | 5.01 | 1.593 | 1.923 |
| Error(b) | 144 | 7493.90 | 52.04 |  |  |  |
| Total | 224 | 40396.92 |  |  |  |  |
| cv(a) %= 18.17 | | cv(b) %= 11.50 | | | | |

Inference: Results indicate the significant difference (@ 5%) among the mean of the three groups of varieties, and significant differences (@ 1%) among the varieties in each of the three groups.

**Table S4: Analysis of Variance (Group Balanced Block Design) for Nodes**

|  | | | | | Tabular F | |
| --- | --- | --- | --- | --- | --- | --- |
| Source of Variation | DF | Sum of Square | MSS | Computed F(b) | 5% | 1% |
| Replication | 2 | 7.25 | 3.62 |  |  |  |
| Varietal Group | 2 | 5.33 | 2.67 | 1.39 | 6.94 | 18.00 |
| Error(a) | 4 | 7.66 | 1.91 |  |  |  |
| Varieties within group A | 24 | 245.22 | 10.22 | 13.94 | 1.593 | 1.923 |
| Varieties within group B | 24 | 160.02 | 6.67 | 9.10 | 1.593 | 1.923 |
| Varieties within group C | 24 | 81.04 | 3.38 | 4.61 | 1.593 | 1.923 |
| Error(b) | 144 | 105.54 | 0.73 |  |  |  |
| Total | 224 | 612.06 |  |  |  |  |
| cv(a) %= 26.97 | | cv(b) %= 16.68 | | | | |

Inference: Results indicate the non significant difference among the mean of the three groups of varieties, but significant differences among the varieties in each of the three groups.

**Table S5: Analysis of Variance (Group Balanced Block Design) for Branches/plant**

|  | | | | | Tabular F |  |
| --- | --- | --- | --- | --- | --- | --- |
| Source of Variation | DF | Sum of Square | MSS | Computed F(b) | 5% | 1% |
| Replication | 2 | 0.13 | 0.07 |  |  |  |
| Varietal Group | 2 | 22.54 | 11.27 | 13.36 | 6.94 | 18.00 |
| Error(a) | 4 | 3.37 | 0.84 |  |  |  |
| Varieties within group A | 24 | 47.42 | 1.98 | 3.36 | 1.593 | 1.923 |
| Varieties within group B | 24 | 37.87 | 1.58 | 2.68 | 1.593 | 1.923 |
| Varieties within group C | 24 | 59.06 | 2.46 | 4.19 | 1.593 | 1.923 |
| Error(b) | 144 | 84.65 | 0.59 |  |  |  |
| Total | 224 | 255.04 |  |  |  |  |
| cv(a) %= 38.51 | | cv(b) %= 32.14 | | | | |

Inference: Results indicate the significant difference (@ 5%) among the mean of the three groups of varieties, but non-significant differences (@ 1%) among the varieties in each of the three groups

**Table S6: Analysis of Variance (Group Balanced Block Design) for Pod**

|  | | | | | Tabular F |  |
| --- | --- | --- | --- | --- | --- | --- |
| Source of Variation | DF | Sum of Square | MSS | Computed F(b) | 5% | 1% |
| Replication | 2 | 331.53 | 165.76 |  |  |  |
| Varietal Group | 2 | 4367.72 | 2183.86 | 11.53 | 6.94 | 18.00 |
| Error(a) | 4 | 757.68 | 189.42 |  |  |  |
| Varieties within group A | 24 | 7281.00 | 303.38 | 5.11 | 1.593 | 1.923 |
| Varieties within group B | 24 | 2568.09 | 107.00 | 1.80 | 1.593 | 1.923 |
| Varieties within group C | 24 | 6676.95 | 278.21 | 4.69 | 1.593 | 1.923 |
| Error(b) | 144 | 8542.29 | 59.32 |  |  |  |
| Total | 224 | 30525.26 |  |  |  |  |
| cv(a) %= 34.67 | | cv(b) %= 19.40 | | |  |  |

**Inference: Results indicate the significant difference (@ 5%) among the mean of the three groups of varieties, and significant differences among the varieties in each of the three groups.**

**Table S7: Analysis of Variance (Group Balanced Block Design) for Biomass data**

|  | | | |  | Tabular F |  |
| --- | --- | --- | --- | --- | --- | --- |
| Source of Variation | DF | Sum of Square | MSS | Computed F(b) | 5% | 1% |
| Replication | 2 | 11593.69 | 5796.84 |  |  |  |
| Varietal Group | 2 | 15299.59 | 7649.79 | 5.66 | 6.94 | 18.00 |
| Error(a) | 4 | 5406.82 | 1351.71 |  |  |  |
| Varieties within group A | 24 | 98001.99 | 4083.42 | 7.84 | 1.593 | 1.923 |
| Varieties within group B | 24 | 50525.58 | 2105.23 | 4.04 | 1.593 | 1.923 |
| Varieties within group C | 24 | 78747.80 | 3281.16 | 6.30 | 1.593 | 1.923 |
| Error(b) | 144 | 74960.36 | 520.56 |  |  |  |
| Total | 224 | 334535.82 |  |  |  |  |
| cv(a) %= 28.94 | | cv(b) %= 17.96 | | |  |  |

**Inference: Results indicate the non significant difference among the mean of the three groups of varieties, but significant differences among the varieties in each of the three groups.**

**Table S8: Analysis of Variance (Group Balanced Block Design) for Seed Yield per plot data**

|  | | | | | Tabular F | |
| --- | --- | --- | --- | --- | --- | --- |
| Source of Variation | DF | Sum of Square | MSS | Computed F(b) | 0.05 | 0.01 |
| Replication | 2 | 290568.2 | 145284.1 |  |  |  |
| Varietal Group | 2 | 5215622 | 2607811 | 66.62924 | 6.94 | 18 |
| Error(a) | 4 | 156556.5 | 39139.13 |  |  |  |
| Varieties within group A | 24 | 18326602 | 763608.4 | 9.455158 | 1.593 | 1.923 |
| Varieties within group B | 24 | 18398659 | 766610.8 | 9.492333 | 1.593 | 1.923 |
| Varieties within group C | 24 | 34603175 | 1441799 | 17.85265 | 1.593 | 1.923 |
| Error(b) | 144 | 11629591 | 80761.05 |  |  |  |
| Total | 224 | 88620773 |  |  |  |  |
| cv(a) %= 12.744 | | cv(b) %= 18.30632 | | |  |  |

**Inference: Results indicate the significant difference among the mean yields of the three groups of varieties, and significant differences among the varieties in each of the three groups.**

**Table S9: Analysis of Variance (Group Balanced Block Design) for Days to Maturity data**

|  | | | | | Tabular F | |
| --- | --- | --- | --- | --- | --- | --- |
| Source of Variation | DF | Sum of Square | MSS | Computed F(b) | 5% | 1% |
| Replication | 2 | 50.96 | 25.48 |  |  |  |
| Varietal Group | 2 | 6022.64 | 3011.32 | 247.23 | 6.94 | 18.00 |
| Error(a) | 4 | 48.72 | 12.18 |  |  |  |
| Varieties within group A | 24 | 176.00 | 7.33 | 1.25 | 1.593 | 1.923 |
| Varieties within group B | 24 | 315.28 | 13.14 | 2.24 | 1.593 | 1.923 |
| Varieties within group C | 24 | 689.52 | 28.73 | 4.89 | 1.593 | 1.923 |
| Error(b) | 144 | 846.32 | 5.88 |  |  |  |
| Total | 224 | 8149.44 |  |  |  |  |
| cv(a) %= 3.58 | | cv(b) %= 2.48 | | | |  |

**Inference: Results indicate the significant difference among the mean of the three groups of varieties, and significant differences among the varieties in group B & C but insignificant in group A.**

**Table S10: Analysis of Variance (Group Balanced Block Design) for 100 Seed Weight data**

|  | | | | | Tabular F | |
| --- | --- | --- | --- | --- | --- | --- |
| Source of Variation | DF | Sum of Square | MSS | Computed F(b) | 5% | 1% |
| Replication | 2 | 51.73 | 25.86 |  |  |  |
| Varietal Group | 2 | 95.66 | 47.83 | 5.87 | 6.94 | 18.00 |
| Error(a) | 4 | 32.62 | 8.15 |  |  |  |
| Varieties within group A | 24 | 114.81 | 4.78 | 3.02 | 1.593 | 1.923 |
| Varieties within group B | 24 | 198.00 | 8.25 | 5.21 | 1.593 | 1.923 |
| Varieties within group C | 24 | 155.07 | 6.46 | 4.08 | 1.593 | 1.923 |
| Error(b) | 144 | 228.22 | 1.58 |  |  |  |
| Total | 224 | 876.11 |  |  |  |  |
| cv(a) %= 20.50 | | cv(b) %= 9.04 | | |  |  |

**Inference: Results indicate the insignificant difference among the mean of the three groups of varieties, and significant differences among the varieties in each group.**

**Table S11: Analysis of Variance (Group Balanced Block Design) for HI data**

|  | | | | | Tabular F | |
| --- | --- | --- | --- | --- | --- | --- |
| Source of Variation | DF | Sum of Square | MSS | Computed F(b) | 5% | 1% |
| Replication | 2 | 467.22 | 233.61 |  |  |  |
| Varietal Group | 2 | 693.63 | 346.82 | 3.86 | 6.94 | 18.00 |
| Error(a) | 4 | 359.54 | 89.88 |  |  |  |
| Varieties within group A | 24 | 2446.20 | 101.92 | 1.27 | 1.593 | 1.923 |
| Varieties within group B | 24 | 3550.28 | 147.93 | 1.85 | 1.593 | 1.923 |
| Varieties within group C | 24 | 4043.29 | 168.47 | 2.10 | 1.593 | 1.923 |
| Error(b) | 144 | 11530.61 | 80.07 |  |  |  |
| Total | 224 | 23090.76 |  |  |  |  |
| cv(a) %= 24.37 | | cv(b) %= 23.00 | | |  |  |

**Inference: Results indicate the insignificant difference among the mean of the three groups of varieties, and insignificant differences among the varieties in group A but significant in group B (@ 5% level) & C (@ 1% Level).**

**Table S12: Analysis of Variance (Group Balanced Block Design) for Yield per plant data**

|  | | | | | Tabular F | |
| --- | --- | --- | --- | --- | --- | --- |
| Source of Variation | DF | Sum of Square | MSS | Computed F(b) | 5% | 1% |
| Replication | 2 | 2245.85 | 1122.92 |  |  |  |
| Varietal Group | 2 | 873.69 | 436.84 | 2.18 | 6.94 | 18.00 |
| Error(a) | 4 | 799.89 | 199.97 |  |  |  |
| Varieties within group A | 24 | 19337.15 | 805.71 | 4.60 | 1.593 | 1.923 |
| Varieties within group B | 24 | 17810.26 | 742.09 | 4.24 | 1.593 | 1.923 |
| Varieties within group C | 24 | 36902.06 | 1537.59 | 8.79 | 1.593 | 1.923 |
| Error(b) | 144 | 25201.51 | 175.01 |  |  |  |
| Total | 224 | 103170.41 |  |  |  |  |
| cv(a) %= 28.55 | | cv(b) %= 26.71 | | |  |  |

**Inference: Results indicate the insignificant difference among the mean yields of the three groups of varieties, but significant differences among the varieties in each of the three groups.**

**Table S13: Genetic variability parameters in across three blocks of advance breeding lines.**

| Parameters | Days to flower | Inflorescence length (cm) | Plant height (cm) | No. of nodes/ Plant | No. of branches  Plant | No. of pods/ Plant | Biomass (gm) | Yield/plot (g) | yield /plant (gm) | Days to maturity | 100-Seed weight (gm) | Harvest Index (%) |
| --- | --- | --- | --- | --- | --- | --- | --- | --- | --- | --- | --- | --- |
| GCV | 13.620 | 37.71 | 17.92 | 27.27 | 31.25 | 21.54 | 23.76 | 36.46 | 33.72 | 5.65 | 10.02 | 11.93 |
| PCV | 14.00 | 43.60 | 21.419 | 32.16 | 44.98 | 29.37 | 30.02 | 40.69 | 43.05 | 6.19 | 13.83 | 25.95 |
| h² (Broad Sense) | 0.94 | 0.74 | 0.70 | 0.71 | 0.48 | 0.53 | 0.62 | 0.80 | 0.61 | 0.83 | 0.52 | 0.212 |
| Genetic Advancement 5% | 10.44 | 1.65 | 19.37 | 2.44 | 1.06 | 12.92 | 49.24 | 1042.62 | 26.94 | 10.37 | 2.086 | 4.40 |
| Mean | 38.28 | 2.46 | 62.72 | 5.13 | 2.38 | 39.70 | 127.03 | 1548.83 | 49.53 | 97.56 | 13.93 | 38.90 |
| Range | 27-48 | 1.23-5.25 | 39.40-102.80 | 2.73-11.60 | 0.48-4.13 | 20.93-70.0 | 66.00-211.67 | 607.67-3833.33 | 22.00-113.58 | 89.00-110.00 | 11.06-18.72 | 25.51-53.67 |

**Table S14**: Mean performance for the 75 advanced breeding for twelve quantitative traits

| **S.No** | **Genotype** | **Days to Flower** | **Inflorescence length (cm)** | **Plant height(cm)** | **Nodes/plant** | **Branch/plant** | **Pods/plant** | **Biomass (g)** | **Yield/plot** | **Yield/plant** | **Days to maturity** | **100 SW (g)** | **HI (%)** |
| --- | --- | --- | --- | --- | --- | --- | --- | --- | --- | --- | --- | --- | --- |
| 1 | **G1** | 34.67 | 1.92 | 72.27 | 7.07 | 1.07 | 33.00 | 84.53 | 2212.33 | 38.23 | 90.00 | 13.97 | 45.11 |
| 2 | **G2** | 33.33 | 5.25 | 55.07 | 4.20 | 2.03 | 35.20 | 150.07 | 2243.33 | 67.87 | 90.00 | 17.87 | 46.22 |
| 3 | **G3** | 36.33 | 1.59 | 75.27 | 7.20 | 2.73 | 49.87 | 187.30 | 1826.33 | 76.40 | 90.00 | 15.37 | 41.11 |
| 4 | **G4** | 32.67 | 2.17 | 60.80 | 7.13 | 0.48 | 30.60 | 83.53 | 1721.33 | 33.60 | 90.67 | 13.57 | 40.04 |
| 5 | **G5** | 33.00 | 1.78 | 67.27 | 6.00 | 2.00 | 36.80 | 126.03 | 1924.00 | 46.63 | 90.33 | 14.13 | 36.72 |
| 6 | **G6** | 31.33 | 3.56 | 49.80 | 3.53 | 1.40 | 20.93 | 75.10 | 2007.67 | 34.80 | 90.67 | 15.03 | 46.34 |
| 7 | **G7** | 34.00 | 3.28 | 50.73 | 3.67 | 2.00 | 32.20 | 130.43 | 2416.67 | 53.03 | 92.33 | 15.17 | 40.18 |
| 8 | **C1** | 31.67 | 2.22 | 41.20 | 3.33 | 1.33 | 22.20 | 66.00 | 2344.33 | 31.20 | 90.00 | 14.13 | 47.29 |
| 9 | **C2** | 30.33 | 4.46 | 41.93 | 2.73 | 2.33 | 24.47 | 71.23 | 1381.67 | 35.63 | 90.00 | 13.57 | 51.41 |
| 10 | **G8** | 32.67 | 2.04 | 58.27 | 4.13 | 0.87 | 27.13 | 100.60 | 1101.00 | 40.63 | 90.33 | 14.17 | 40.33 |
| 11 | **G9** | 34.00 | 4.45 | 62.80 | 4.60 | 1.47 | 24.53 | 79.90 | 1653.67 | 26.97 | 91.33 | 13.83 | 33.99 |
| 12 | **G10** | 39.00 | 1.26 | 67.53 | 5.67 | 2.80 | 47.53 | 150.30 | 1284.33 | 43.50 | 91.00 | 13.70 | 30.76 |
| 13 | **G11** | 40.00 | 1.24 | 69.27 | 5.47 | 2.93 | 49.07 | 149.93 | 1395.67 | 48.73 | 91.00 | 13.40 | 32.17 |
| 14 | **G12** | 34.33 | 1.71 | 44.73 | 4.47 | 2.07 | 32.73 | 129.60 | 2175.33 | 58.23 | 97.00 | 15.67 | 44.79 |
| 15 | **G13** | 35.67 | 2.70 | 60.87 | 4.80 | 2.13 | 51.13 | 122.80 | 868.67 | 39.37 | 90.00 | 13.47 | 32.23 |
| 16 | **G14** | 38.67 | 1.73 | 100.80 | 11.60 | 1.33 | 41.67 | 124.73 | 1224.67 | 44.87 | 93.33 | 13.17 | 36.26 |
| 17 | **G15** | 35.33 | 2.00 | 52.73 | 4.47 | 2.93 | 40.33 | 137.40 | 1300.33 | 54.40 | 90.00 | 16.10 | 39.02 |
| 18 | **G16** | 34.33 | 1.96 | 70.67 | 6.80 | 0.53 | 30.13 | 94.67 | 2246.67 | 49.30 | 90.00 | 13.77 | 51.80 |
| 19 | **G17** | 30.00 | 4.43 | 51.68 | 3.85 | 1.57 | 26.43 | 75.70 | 1443.67 | 32.30 | 89.00 | 13.93 | 43.13 |
| 20 | **G18** | 27.33 | 2.31 | 61.33 | 5.47 | 1.47 | 24.00 | 68.33 | 607.67 | 25.07 | 90.00 | 13.23 | 36.12 |
| 21 | **G19** | 37.00 | 3.52 | 60.20 | 4.80 | 2.17 | 47.60 | 153.57 | 1683.67 | 65.00 | 90.00 | 14.30 | 41.00 |
| 22 | **G20** | 34.00 | 2.23 | 53.07 | 4.33 | 1.87 | 28.27 | 120.87 | 2470.33 | 55.53 | 90.33 | 15.97 | 45.93 |
| 23 | **G21** | 34.67 | 2.13 | 48.47 | 4.00 | 3.73 | 55.27 | 191.67 | 2236.00 | 93.93 | 90.67 | 16.17 | 48.52 |
| 24 | **G22** | 34.00 | 4.72 | 55.27 | 3.93 | 3.07 | 34.20 | 158.73 | 2054.33 | 66.03 | 90.33 | 16.83 | 42.02 |
| 25 | **G23** | 34.67 | 2.38 | 55.47 | 3.73 | 2.20 | 33.87 | 113.30 | 1943.33 | 45.83 | 90.00 | 15.80 | 40.50 |
| 26 | **C3** | 41.00 | 3.27 | 63.30 | 5.67 | 1.67 | 29.07 | 98.17 | 1087.67 | 40.40 | 97.67 | 13.37 | 41.16 |
| 27 | **G24** | 36.33 | 1.97 | 74.95 | 5.35 | 3.47 | 47.17 | 140.30 | 1647.00 | 48.13 | 96.67 | 13.33 | 34.72 |
| 28 | **G25** | 44.67 | 1.31 | 72.73 | 6.13 | 3.53 | 46.27 | 144.87 | 967.00 | 52.17 | 98.00 | 13.77 | 35.88 |
| 29 | **C4** | 42.33 | 2.46 | 74.73 | 4.33 | 2.53 | 35.33 | 113.77 | 870.67 | 42.23 | 98.33 | 12.47 | 37.20 |
| 30 | **G26** | 32.67 | 1.25 | 102.80 | 10.73 | 1.07 | 46.07 | 152.67 | 904.00 | 45.33 | 98.33 | 14.67 | 29.66 |
| 31 | **G27** | 34.67 | 2.07 | 46.47 | 3.47 | 2.40 | 36.73 | 140.67 | 2194.00 | 67.93 | 100.00 | 15.37 | 47.57 |
| 32 | **G28** | 30.67 | 3.75 | 39.40 | 3.60 | 4.13 | 30.22 | 81.63 | 1210.33 | 41.70 | 96.67 | 13.90 | 48.70 |
| 33 | **G29** | 34.00 | 4.14 | 59.00 | 5.93 | 2.13 | 42.03 | 127.54 | 1111.33 | 46.65 | 97.00 | 13.67 | 37.93 |
| 34 | **G30** | 31.67 | 3.57 | 53.47 | 5.85 | 2.00 | 45.97 | 108.53 | 1230.00 | 38.10 | 100.33 | 11.93 | 35.02 |
| 35 | **G31** | 36.33 | 2.66 | 48.53 | 4.87 | 2.60 | 40.07 | 159.73 | 2191.00 | 76.23 | 98.67 | 15.63 | 48.07 |
| 36 | **G32** | 34.00 | 2.39 | 55.80 | 4.80 | 3.07 | 46.00 | 173.10 | 1779.33 | 76.80 | 96.67 | 18.23 | 42.99 |
| 37 | **G33** | 34.33 | 1.65 | 69.67 | 5.87 | 2.07 | 44.33 | 137.40 | 1915.00 | 52.20 | 100.00 | 13.00 | 38.08 |
| 38 | **G34** | 32.00 | 3.08 | 48.53 | 3.60 | 3.67 | 34.73 | 106.37 | 1477.33 | 38.03 | 97.33 | 13.53 | 35.73 |
| 39 | **G35** | 32.00 | 3.20 | 46.87 | 3.33 | 2.93 | 31.00 | 104.90 | 1058.00 | 27.30 | 97.33 | 15.10 | 27.02 |
| 40 | **G36** | 38.00 | 2.15 | 67.67 | 5.33 | 1.87 | 38.60 | 116.17 | 939.33 | 32.63 | 96.33 | 13.47 | 28.05 |
| 41 | **G37** | 33.33 | 3.54 | 57.87 | 4.40 | 2.33 | 29.60 | 114.93 | 2094.67 | 52.07 | 97.00 | 14.87 | 45.57 |
| 42 | **G38** | 31.67 | 3.46 | 53.60 | 4.33 | 2.20 | 39.87 | 188.97 | 1942.33 | 83.97 | 97.33 | 18.72 | 44.02 |
| 43 | **G39** | 43.00 | 1.53 | 70.00 | 4.40 | 3.67 | 36.87 | 85.37 | 706.00 | 23.30 | 103.67 | 12.03 | 30.16 |
| 44 | **G40** | 34.00 | 2.31 | 55.40 | 4.13 | 2.87 | 29.87 | 94.10 | 1931.00 | 31.07 | 96.33 | 13.83 | 34.06 |
| 45 | **G41** | 42.00 | 2.16 | 61.82 | 4.20 | 3.40 | 46.07 | 135.70 | 759.00 | 34.32 | 103.33 | 11.68 | 25.88 |
| 46 | **G42** | 36.33 | 3.10 | 57.27 | 5.47 | 2.00 | 35.40 | 138.23 | 2230.00 | 64.87 | 99.33 | 13.97 | 47.10 |
| 47 | **G43** | 42.00 | 1.70 | 63.87 | 3.93 | 2.93 | 34.67 | 128.97 | 1212.33 | 44.40 | 98.67 | 13.70 | 34.39 |
| 48 | **G44** | 42.00 | 1.37 | 58.23 | 4.53 | 2.33 | 38.47 | 128.23 | 1636.67 | 57.52 | 100.67 | 13.33 | 48.23 |
| 49 | **G45** | 44.33 | 1.31 | 66.00 | 3.93 | 3.07 | 33.73 | 103.50 | 841.00 | 41.70 | 102.00 | 13.83 | 40.11 |
| 50 | **C5** | 41.67 | 1.82 | 69.87 | 6.17 | 2.23 | 38.50 | 110.80 | 1138.33 | 40.63 | 99.67 | 14.10 | 37.22 |
| 51 | **G46** | 46.33 | 1.33 | 72.53 | 3.47 | 3.07 | 31.27 | 132.73 | 1780.67 | 45.95 | 106.00 | 17.30 | 34.57 |
| 52 | **C6** | 48.33 | 4.29 | 86.27 | 6.27 | 2.73 | 60.67 | 147.07 | 1555.33 | 51.50 | 110.00 | 12.37 | 35.00 |
| 53 | **G47** | 46.33 | 1.88 | 75.40 | 6.67 | 0.93 | 44.87 | 141.37 | 2155.67 | 52.03 | 107.33 | 13.07 | 37.43 |
| 54 | **G48** | 41.33 | 1.54 | 77.00 | 6.67 | 1.73 | 44.13 | 84.97 | 1005.67 | 42.20 | 98.67 | 12.67 | 50.23 |
| 55 | **G49** | 43.33 | 1.23 | 74.20 | 5.80 | 2.20 | 50.53 | 130.50 | 1141.00 | 45.23 | 102.67 | 12.87 | 34.56 |
| 56 | **G50** | 43.00 | 1.57 | 61.60 | 5.27 | 2.73 | 59.00 | 149.77 | 1476.67 | 59.73 | 100.67 | 12.27 | 38.76 |
| 57 | **G51** | 42.67 | 2.61 | 55.03 | 4.33 | 2.37 | 56.43 | 147.90 | 1906.00 | 58.43 | 100.67 | 12.97 | 38.94 |
| 58 | **G52** | 42.67 | 1.40 | 71.40 | 5.27 | 2.93 | 51.13 | 132.37 | 619.00 | 37.57 | 102.33 | 11.62 | 28.85 |
| 59 | **G53** | 47.00 | 1.65 | 63.00 | 4.73 | 3.07 | 49.67 | 138.13 | 1295.00 | 48.50 | 102.33 | 12.73 | 35.03 |
| 60 | **G54** | 45.67 | 3.79 | 81.00 | 8.00 | 3.90 | 70.00 | 211.67 | 3833.33 | 113.58 | 106.67 | 13.03 | 53.67 |
| 61 | **G55** | 41.00 | 3.26 | 62.52 | 4.88 | 2.58 | 43.05 | 126.82 | 1175.67 | 45.30 | 100.00 | 13.77 | 35.75 |
| 62 | **G56** | 47.00 | 1.73 | 71.60 | 6.80 | 0.73 | 40.87 | 131.73 | 974.00 | 45.50 | 101.33 | 14.40 | 34.56 |
| 63 | **G57** | 39.33 | 2.93 | 60.07 | 4.80 | 3.33 | 52.00 | 194.40 | 2359.33 | 96.70 | 98.00 | 15.90 | 49.55 |
| 64 | **G58** | 42.67 | 1.77 | 54.47 | 4.47 | 2.07 | 31.73 | 97.83 | 1424.67 | 36.10 | 102.33 | 13.00 | 37.44 |
| 65 | **G59** | 42.67 | 1.40 | 76.73 | 5.80 | 2.60 | 58.27 | 176.47 | 1562.33 | 55.63 | 99.00 | 13.63 | 31.68 |
| 66 | **G60** | 43.00 | 2.59 | 52.13 | 4.73 | 2.13 | 41.47 | 163.10 | 2165.67 | 75.47 | 100.67 | 13.47 | 45.52 |
| 67 | **G61** | 42.67 | 2.33 | 64.80 | 6.07 | 1.07 | 36.93 | 149.13 | 1713.33 | 61.40 | 103.67 | 13.73 | 40.97 |
| 68 | **G62** | 42.33 | 2.14 | 55.53 | 4.60 | 3.87 | 45.73 | 202.40 | 1882.00 | 97.67 | 103.00 | 15.34 | 48.09 |
| 69 | **G63** | 42.67 | 2.74 | 60.80 | 4.87 | 2.47 | 40.07 | 114.20 | 866.33 | 44.17 | 102.67 | 12.37 | 39.21 |
| 70 | **G64** | 44.33 | 1.59 | 65.73 | 5.27 | 2.00 | 38.60 | 134.77 | 985.00 | 45.40 | 106.00 | 11.45 | 34.57 |
| 71 | **G65** | 47.00 | 1.66 | 76.32 | 6.50 | 2.00 | 41.27 | 118.17 | 970.00 | 30.60 | 106.00 | 11.50 | 25.87 |
| 72 | **G66** | 42.00 | 2.56 | 58.88 | 4.47 | 3.53 | 37.40 | 106.40 | 1210.00 | 28.80 | 103.33 | 11.06 | 28.90 |
| 73 | **G67** | 41.67 | 1.93 | 62.33 | 4.37 | 3.60 | 37.53 | 96.73 | 760.33 | 22.00 | 105.33 | 12.53 | 25.51 |
| 74 | **G68** | 42.33 | 2.38 | 61.73 | 3.90 | 4.13 | 37.53 | 88.20 | 637.33 | 22.00 | 104.67 | 12.60 | 28.90 |
| 75 | **C7** | 46.00 | 3.67 | 77.83 | 5.53 | 2.37 | 41.57 | 129.43 | 1867.67 | 46.23 | 108.00 | 11.13 | 36.53 |

Table S 15: Cluster Distances (intra and inter) among the advanced breeding lines

|  | Cluster I | Cluster II | Cluster III | Cluster IV | Cluster V |
| --- | --- | --- | --- | --- | --- |
| Cluster I | 8.04 | 10.79 | 12.37 | 10.85 | 14.86 |
| Cluster II |  | 6.79 | 18.24 | 11.66 | 19.76 |
| Cluster III |  |  | 0.00 | 15.57 | 10.17 |
| Cluster IV |  |  |  | 6.17 | 17.33 |
| Cluster V |  |  |  |  | 0.00 |

Table S 16: Cluster means for the 12 quantitative traits in advanced breeding lines

|  | DF | IL | PH | NP | BP | PP | BM | PY | YP | DM | 100 SW | HI |
| --- | --- | --- | --- | --- | --- | --- | --- | --- | --- | --- | --- | --- |
| Cluster I | 39.70 | 2.17 | 63.19 | 5.06 | 2.50 | 41.09 | 131.19 | 1520.10 | 50.42 | 98.50 | 13.78 | 38.27 |
| Cluster II | 31.67 | 3.54 | 52.25 | 4.29 | 1.95 | 29.77 | 100.95 | 1571.38 | 41.80 | 92.43 | 14.70 | 41.55 |
| Cluster III | 48.33 | 4.29 | 86.27 | 6.27 | 2.73 | 60.67 | 147.07 | 1555.33 | 51.50 | 110.00 | 12.37 | 35.00 |
| Cluster IV | 35.67 | 1.49 | 101.80 | 11.17 | 1.20 | 43.87 | 138.70 | 1064.33 | 45.10 | 95.83 | 13.92 | 32.96 |
| Cluster V | 45.67 | 3.79 | 81.00 | 8.00 | 3.90 | 70.00 | 211.67 | 3833.33 | 113.58 | 106.67 | 13.03 | 53.67 |

**^#^**DF-Days to flowering, IL-Inflorescence length, PH-Plant height, NP-Nodes per plant, BP-Branches per plant, PP-Pods per plant, BM-biomass/plant, PY-yield/plot, YP-yield per plant, DM-Days to maturity, 100 SW-100 seed weight, HI-Harvest index

**Table S17: Multi-location evaluation of NRC 128 for grain yield (kg/ha) in Northern Plain Zone (three locations) of India**

| S. No | Genotype | Delhi | Ludhiana | Pantnagar | Mean |
| --- | --- | --- | --- | --- | --- |
| L1 | PS 1613 | 2407.40^a^ | 1083.33^b^ | 2604.16^a^ | 2031.63^ab^ |
| L2 | PS 1611 | 1857.63^b^ | 751.15^cd^ | 1655.09^b^ | 1421.29^cd^ |
| L3 | NRC 128 | 2314.81^a^ | 1910.88^a^ | 2500.00^a^ | 2241.89^a^ |
| L4 | PS 1347(C) | 1562.50^c^ | 2118.05^a^ | 1666.66^b^ | 1782.40^bc^ |
| L5 | Pusa 97-12(C) | 912.03^e^ | 520.83^d^ | 1516.20^bc^ | 983.024^e^ |
| L6 | SL 958(C) | 1261.57^d^ | 943.28^bc^ | 1400.46^c^ | 1201.77^de^ |

**Table S18: Multi-location evaluation of NRC 128 grain yield (kg/ha) in Eastern Zone (Four locations) of India.**

| S. No | Genotype | Bhawanipatna | Dholi | Raipur | Ranchi | Mean |
| --- | --- | --- | --- | --- | --- | --- |
| L1 | RSC 11-07 | 1611.11^a^ | 1134.26^cd^ | 1927.08^a^ | 2349.54^a-d^ | 1755.50^a^ |
| L2 | NRC 128 | 1562.50^ab^ | 1365.74^a^ | 1892.36^a^ | 2060.19^de^ | 1720.20^ab^ |
| L3 | AMS 2014-1 | 1399.31^bcd^ | 891.20^gh^ | 1649.31^bc^ | 2584.49^a^ | 1631.08^abc^ |
| L4 | NEC 136 | 1250.00^def^ | 1087.96^cde^ | 1035.88^fg^ | 2413.19^abc^ | 1446.76^a-d^ |
| L5 | MACS 1493 | 1680.56^a^ | 1064.81^c-f^ | 914.35^g^ | 2447.92^ab^ | 1526.91^a-d^ |
| L6 | RSC 11-03 | 1210.65^d-g^ | 925.93^fgh^ | 1539.35^cd^ | 1848.38^e^ | 1381.08^bcd^ |
| L7 | NRCSL 1 | 1497.69^abc^ | 1317.13^ab^ | 1817.13^ab^ | 2089.12^de^ | 1680.27^abc^ |
| L8 | NRC 132 | 1343.75^c-f^ | 1030.09^d-g^ | 1203.70^ef^ | 2604.17^a^ | 1545.43^a-d^ |
| L9 | NRC 137 | 1199.07^efg^ | 879.63^h^ | 1186.34^ef^ | 2118.06^cde^ | 1345.78^cd^ |
| L10 | NRC 147 | 1385.42^b-e^ | 914.35^gh^ | 1006.94^g^ | 1510.42^f^ | 1204.28^d^ |
| L11 | JS 335(C) | 1175.93^fg^ | 972.22^e-h^ | 1215.28^e^ | 2214.12^bcd^ | 1394.39^bcd^ |
| L12 | RKS 18(C) | 1226.85^d-g^ | 1116.90^cd^ | 1192.13^ef^ | 2239.58^bcd^ | 1443.87^a-d^ |
| L13 | JS 97-52(C) | 1037.04^g^ | 1180.56^bc^ | 1429.40^d^ | 2563.66^a^ | 1552.66^a-d^ |

**Table S19: Pooled analysis of variance for genotypes evaluated across two agro-climatic Zones.**

| Eastern Zone | | | Northern Plain Zone | | |
| --- | --- | --- | --- | --- | --- |
| Source of variation | D.f | F value | Source of variation | D.f | F value |
| Environment | 3 | 866.18^***^ | Environment | 2 | 50.29^***^ |
| Replication (Environment) | 12 | 0.687^NS^ | Replication (Environment) | 9 | 3.83^**^ |
| Genotype | 12 | 18.68^***^ | Genotype | 5 | 191.65^***^ |
| Genotype x Environment | 36 | 10.79^***^ | Genotype x Environment | 10 | 42.00^***^ |
| Residuals | 144 | - | Residuals | 45 | - |
| CV(%) | 9.85 | - | CV(%) | 7.62 | - |

^***^p<0.001, ^**^p<0.01and ^NS^non-significant

**Fig. S1 Clustering of soybean genotypes by Tocher’s method**

**
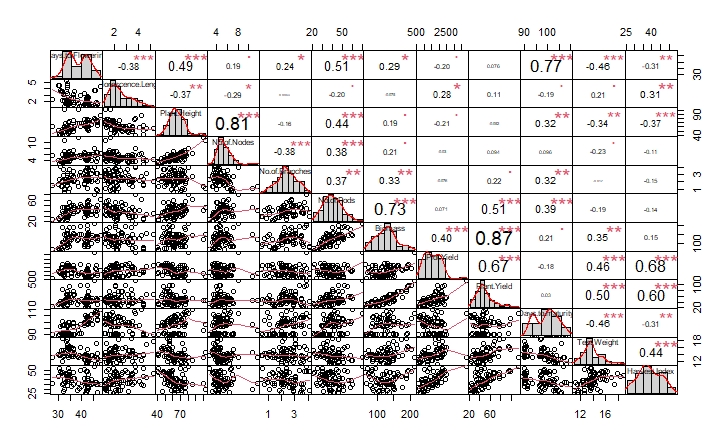
**

**Fig S2. Correlation analysis of twelve quantitative traits in 75 breeding lines** (Generated using R package“PerformanceAnalytics version 2.0.4 URL https://github.com/braverock/PerformanceAnalytics**)**
